# Interferon Receptor Chain Deficiency in Murine Friend Erythroleukemia Cell Clone Resistant to Type I or Type I and II Interferons

**DOI:** 10.3390/ijms27135908

**Published:** 2026-06-30

**Authors:** Zulema Antonia Percario, Giorgio Mangino, Arianna Raponi, Emiliano Fratini, Gabriele Vaccari, Flavia Giannessi, Gianna Fiorucci, Manuela Cervelli, Giovanna Romeo, Elisabetta Affabris

**Affiliations:** 1 Department of Science, Roma Tre University, 00146 Rome, Italy; zulema.percario@uniroma3.it (Z.A.P.); arianna.raponi@glasgow.ac.uk (A.R.); flaviagiannessi@gmail.com (F.G.); manuela.cervelli@uniroma3.it (M.C.); 2 Department of Medico-Surgical Sciences and Biotechnologies, Sapienza University of Rome, 04100 Latina, Italy; giorgio.mangino@uniroma1.it (G.M.); giovanna.romeo@uniroma1.it (G.R.); 3 Laboratory of Red Biotechnologies, Italian National Agency for New Technologies, Energy and Sustainable Economic Development (ENEA), Casaccia Research Centre, 00123 Rome, Italy; emiliano.fratini@enea.it; 4 Department of Food Safety and Veterinary Public Health, Istituto Superiore di Sanità, 00161 Rome, Italy; gabriele.vaccari@iss.it; 5 Institute of Molecular Biology and Pathology, Consiglio Nazionale delle Ricerche, 00185 Rome, Italy; fioruccigianna@gmail.com

**Keywords:** type I IFN, type II IFN, type I IFN receptors, type II IFN receptor, murine IFNAR, murine IFNGR, Friend erythroleukemia cells, murine retrovirus, Friend erythroleukemia virus

## Abstract

Interferon (IFN)-resistant cell clones 3Cl8 and 3γR8, isolated from wild-type Friend erythroleukemia cells 745A were characterized to identify the resistance defect. The 3Cl8 cell clone is resistant to type I IFNs and sensitive to type II IFN, whereas 3γR8, derived from 3Cl8, is resistant to both type I and II IFNs. Here, we report that no activation of the JAK-STAT pathway is detected after IFN treatment of resistant cells. Interestingly, the absence of major transcripts of the IFNAR2 receptor chain has been observed in type I IFN-resistant cells, and a point mutation relative to the IFNGR2 receptor chain (β chain) has been identified in type II IFN-resistant cells, inducing a frameshift leading to premature termination of translation. In addition, we have identified a new polymorphism of the murine IFNAR1 chain and possibly the presence of a murine IFNAR2b transmembrane, non-transducing chain in 745A cells, similar to that observed in humans and differing from previous reports on other murine systems.

## 1. Introduction

Friend leukemia cells (FLCs) are murine (mu) tumor cells derived from DBA-2 mice chronically infected with the Friend erythroleukemia retroviral complex, isolated by Charlotte Friend in 1957 [1] and known as Friend Leukemia Virus (FLV). The FLC clones used in various laboratories originate from transplantable tumors arising in leukemic mice inoculated with FLV [2,3]. FLC clones are blocked in their differentiation potential at the proerythroblast stage and do not produce hemoglobin or globin mRNA, but they can differentiate when treated with DMSO or other differentiation inducers [4,5,6,7].

Interferons (IFNs) are species-specific cytokines subdivided into three classes based on their cell membrane receptors and antigenic relationships, exerting antimicrobial, antiproliferative, differentiative, and immunomodulatory effects on susceptible target cells [8,9,10]. All type I IFNs (α_s_, β, ω, κ, ε and τ subtypes) use a heterodimeric receptor composed of the IFNAR1 and IFNAR2 subunits, whereas type II IFN (IFN-γ) and type III IFNs (λ-1, -2 and -3 in the murine system) use receptors formed by IFNGR1 + IFNGR2 and IFNLR1 + IL10R2 subunits, respectively [8,11,12,13,14,15,16,17,18]. The interaction of IFNs with their receptors activates the Janus Kinases (JAK)-Signal Transducers and Activators of Transcription (STAT) pathway, stimulating the transcription of a set of immediate-early genes known as IFN-sensitive genes (ISGs). The JAK-STAT pathway involves activation of specific cytoplasmic STAT proteins via non-receptor tyrosine kinases JAK associated with the IFN receptor subunits [19,20]. Tyrosine-phosphorylated STATs form homo- or heterodimers, which translocate into the nucleus to regulate gene expression [21,22]. In type I IFN-sensitive cells, ISG regulation by IFN-α or -β primarily involves the transcription factor ISGF3 (STAT-1/STAT-2/IRF9), which acts on ISRE-regulated genes, and the GAF transcription factor (STAT1 homodimer) that is formed to a lesser extent. In type II-sensitive cells, ISG regulation by IFN-γ mainly involves STAT1 activation, forming the GAF transcription factor that activates GAS-regulated genes including the transcription factor IRF-1. In turn, IRF-1 also activates ISRE-regulated genes [20,21].

The engagement of the IFN receptor can also activate additional signaling pathways, such as MAPK (i.e., p38 and ERK), NF-κB, and PI3K/AKT, to optimize ISG transcription and biological responses. The contribution of these pathways varies depending on cell type and context [23].

Mu IFN-α and -β induce a potent antiviral state in FLC, blocking chronic FLV release in a reversible manner, thereby containing viral replication and tumor growth in vivo [24,25,26,27,28,29]. They also modulate FLC erythroid differentiation and inhibit cell growth in vitro [30,31,32]. In 1982, FLC clones resistant to type I IFN were isolated by cultivating the FLC clone 745A—originally provided by Charlotte Friend—for more than four months in the continuous presence of an increasing amounts of mu IFN-αβ, a mixture of the α and β types [33]. The 3Cl8 clone, initially named MRI 745 3Cl8, was completely resistant to the antiviral and antiproliferative effects of IFN-αβ, still retaining the ability to bind type I IFN to its membrane, which suggests the presence of functional receptor chains [34]. This clone possesses full susceptibility to IFN-γ, although it responds less than wild-type 745A cells to the antiviral and antiproliferative effects of IFN-γ, since IFN-γ treatment induces the production of IFN-β in wt 745A cells through a post-transcriptional mechanism [35]. The produced IFN-β acts synergistically with IFN-γ, thereby reinforcing the physiological response and mounting a very strong antiviral state in wt cells [35]. 3Cl8 cells respond to IFN-γ without the synergistic effect observed between type I and type II IFNs [35,36,37].

In 1988, FLC clones with an IFN-γ-resistant phenotype were isolated [38]. These clones were selected through prolonged cultivation of wt 745A cells or the IFN-αβ-resistant clone 3Cl8 in the presence of increasing amounts of highly purified murine recombinant (mu rec) IFN-γ, aimed at progressively inhibiting cell growth. Clones fully resistant to the antiviral and antiproliferative effects of IFN-γ were obtained only from cells already resistant to IFN-αβ, whereas clones derived from wild-type 745A retained partial susceptibility to IFN-γ [38]. The clone, named 3γR8 (combining ‘3’ from 3Cl8, ‘γR’ from IFN γ-resistant, and ‘8’ from clone 8), as well as its subclone ABGR (α-, β-and γ-resistant), was further characterized. These cells are fully resistant to both IFN-αβ and IFN-γ. They can bind IFN-γ but do not exhibit induction of ISGs such as 2-5A synthetase and PKR after treatment with IFN-αβ or IFN-γ [38]. Band-shift assays assessing the formation of DNA–protein complexes at the interferon-stimulated response element (ISRE) of the 2-5A synthetase gene promoter revealed a lack of complex formation in 3Cl8 cells treated with IFN-αβ and in 3γR8 and ABGR cells treated with IFN-αβ or IFN-γ [39]. These findings indicate that the IFN-resistant phenotype results from defects between the binding of IFN to its receptor and subsequent formation and activation of ISGF3 (STAT1-STAT2-IRF9 complex) and/or GAF transcription factors, which are essential for type I and II IFN-mediated activation of ISGs, respectively [39,40,41,42].

Here, we have analyzed the activation of the JAK-STAT pathways in IFN-sensitive and resistant FLC clones, as well as the expression of IFNAR (type I IFN receptor) and IFNGR (type II IFN receptor) subunits, identifying defects at the level of one of the receptor subunits of each receptor. Additionally, we have identified a new polymorphism in the murine IFNAR1 chain in FLC and hypothesized the existence of a non-transducing mu IFNAR2b transmembrane chain in these cells, similar to that identified in humans but not yet described in mice.

## 2. Results

### 2.1. JAK-STAT Signal Transduction Pathway and STAT-1, -2 and -3 Expressions in wt 745A, 3Cl8 and 3γR8/ABGR FLC Treated with Type I or II IFNs

The binding of type I IFNs to their receptor chains induces the tyrosine phosphorylation and activation of the JAK tyrosine kinases Tyk-2 and JAK1 in sensitive cells, resulting in the tyrosine phosphorylation of STAT1α, STAT1β and STAT2; their heterodimerization; and their association with the IRF family protein IRF9 to form the heterotrimeric transcription factor ISGF3 (STAT1-STAT2-IRF9). Conversely, the binding of IFN-γ to its receptor induces the tyrosine phosphorylation and activation of JAK1 and JAK2, which in turn phosphorylate STAT1 at tyrosine 701, leading to the formation of the transcription factor GAF. GAF is also formed, at lower efficiency and transiently, during type-I IFN signaling due to STAT1 homodimerization in addition to its predominant heterodimerization with STAT2 [43,44,45,46,47]. Tyrosine phosphorylation and activation of other STAT family members by type I or II IFNs have been described in some cell types, with STAT3 activation by IFN-α or IFN-γ being the most frequently reported [48,49,50,51].

**Figure 1:**
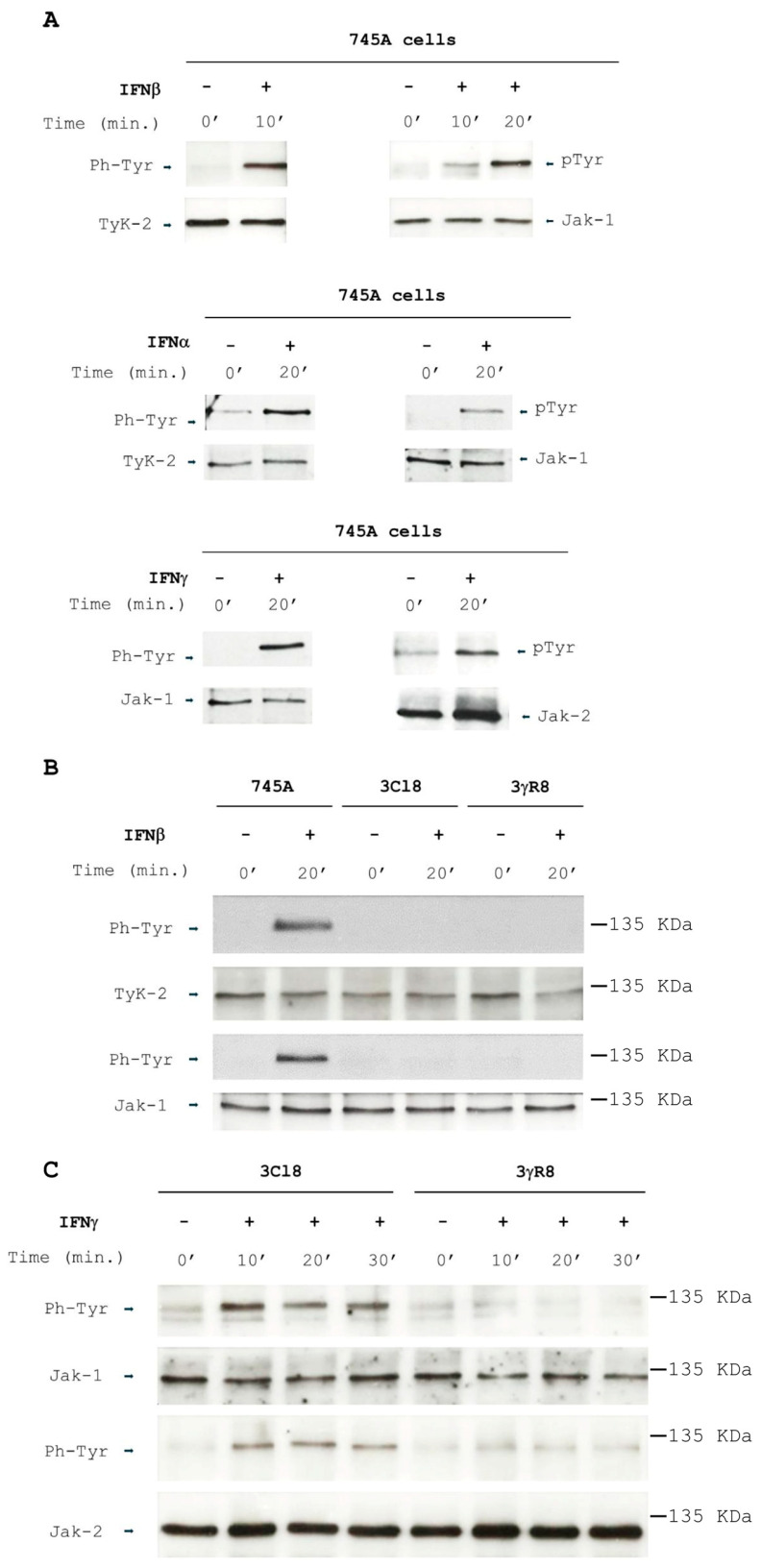
Tyk2, JAK 1 and JAK 2 tyrosine phosphorylation in IFN-sensitive and resistant FLCs upon treatment with mu IFN-α, -β or -γ. (**A**) 745A cells (2.5 × 10^6^ cells/mL) were treated with 500 IU/mL IFN-α, -β or -γ for the indicated times. (**B**,**C**) 745A, 3Cl8 or 3γR8 cells (2.5 × 10^6^ cells/mL) were treated with 500 IU/mL IFN-β or -γ for the indicated times. Immunoprecipitation followed by Western blot analysis was performed as described in Section 4 (Materials and Methods). Immunoblotting was first performed with an anti-phospho-tyrosine monoclonal antibody (anti-P-Tyr-100). Membranes were then reprobed with anti-Tyk2, -JAK1 or -JAK2 antibodies after stripping. One representative experiment is shown (for densitometric quantification, see Supplementary Materials, Figures S5–S7).

**Figure 2:**
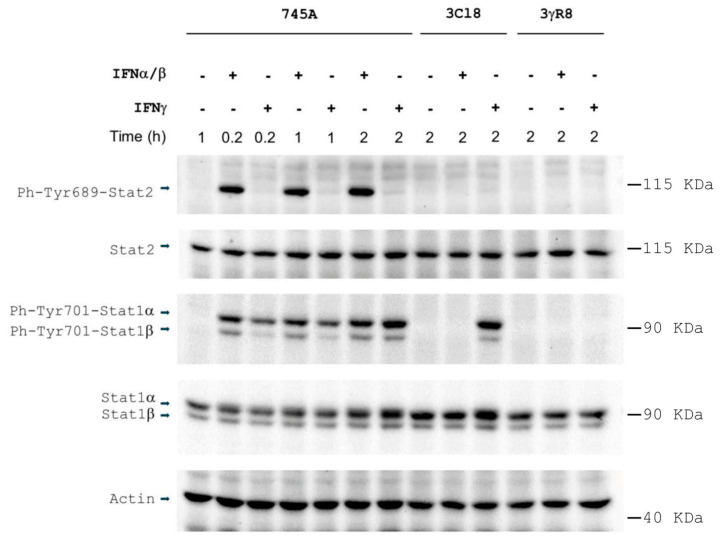
STAT1 and STAT2 tyrosine phosphorylation induced by treatment of wt and resistant FLC clones with IFN-α/β or -γ. A total of 2.5 × 10^6^ cells/mL were treated with 500 IU/mL mu IFN-α/β or -γ for the indicated times. Western blot analysis was performed as described in Section 4 (Materials and Methods). Immunoblotting was first performed with anti-phospho-Tyr689-Stat2 or anti-phospho-Tyr701-Stat1 antibodies. After stripping, membranes were reprobed with anti-Stat2 or anti-Stat1 antibodies. As a loading control, membranes were incubated with an anti-Actin antibody. One representative experiment is shown (for densitometric quantification, see Supplementary Materials, Figure S8).

Figure 1A shows the tyrosine phosphorylation of Tyk2 and JAK1 after 10–20 min of wt 745A cells treatment with mu IFN-α or -β, and it shows the tyrosine phosphorylation of JAK1 and JAK2 after 20 min of treatment with mu IFN-γ. Treatment of 3Cl8 or 3γR8 cells with IFN-β does not induce tyrosine phosphorylation of Tyk2 and JAK1, whereas treatment with IFN-γ induces tyrosine phosphorylation of JAK1 and JAK2 only in the 3Cl8 clone (Figure 1B and Figure 1C, respectively). Accordingly, IFN-β does not induce STAT1 and STAT2 tyrosine phosphorylation in 3Cl8 and 3γR8 cells, while IFN-γ induces STAT1 tyrosine phosphorylation only in IFN-γ–sensitive cells (i.e., wt 745A and 3Cl8) (Figure 2).

**Figure 3:**
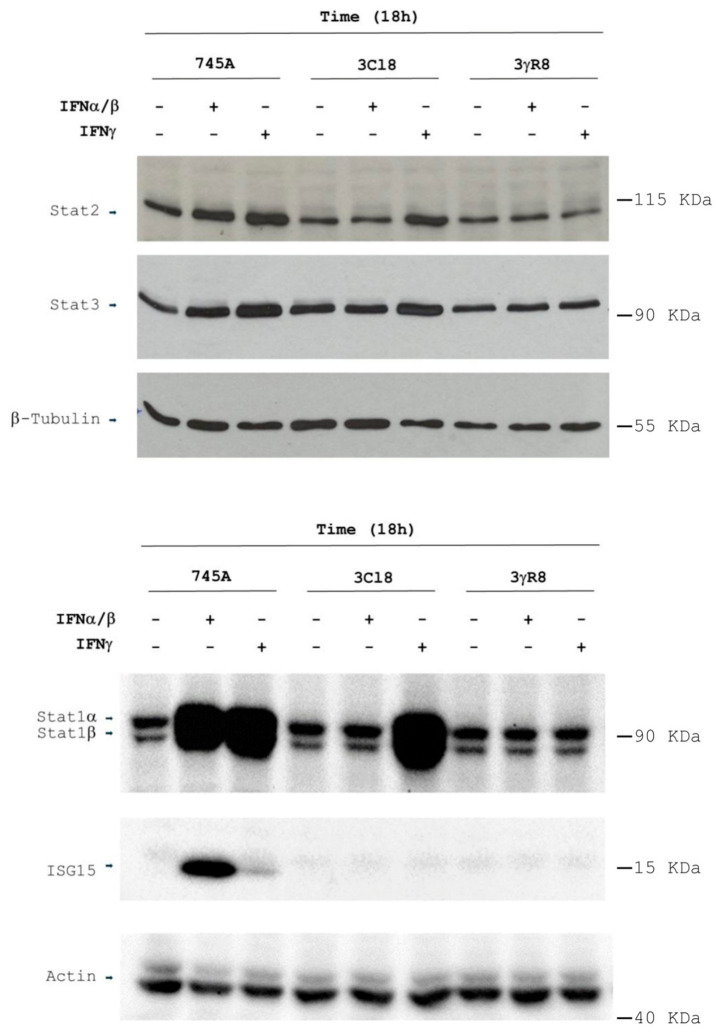
Expression of STAT1, STAT2, STAT3 and ISG15 in 745A, 3Cl8 and 3γR8 cell clones treated for 18 h with mu IFN-α/β or -γ. Cells were seeded at 1.5 × 10^6^ cells/mL and treated with 500 IU/mL IFN-α/β or IFN-γ. Western blot analysis was performed as described in Section 4 (Materials and Methods). Immunoblotting was performed with anti-Stat1, anti-Stat2, anti-Stat3 or anti-ISG15 antibodies. As a loading control, membranes were incubated with anti β-Tubulin or anti-actin antibodies. One representative experiment is shown (for densitometric quantification, see Supplementary Materials, Figure S9).

Some STATs are themselves ISGs, and their IFN-dependent transcriptional increase relies on the presence of ISRE and GAS enhancers in their promoters [21]. Supplementary Figure S1 shows the time-dependent upregulation of STAT1 and STAT2 in wt 745A cells treated with mu IFN-β or mu IFN-γ, respectively. Figure 3 shows the increase in the expression of STAT1, -2, and -3 after 18 h of wt cells treatment with IFN-β or IFN-γ. No induction of these proteins was observed in 3γR8 cells, while only IFN-γ increased the expression of STATs in 3Cl8 cells (Figure 3). ISG15, a ubiquitin-like protein induced by type I, but not by type II IFNs [52,53], is upregulated in wt 745A FLC treated with IFN-β but not in 3Cl8 or 3γR8 cells (Figure 3). In conclusion, the analysis of the JAK-STAT pathway indicates that the IFN-α/β and IFN-γ resistant phenotypes are due to defects between the IFN receptor binding and JAK kinase activation.

To determine whether the type I IFN-resistant phenotype is dominant or recessive, we performed somatic cell hybridization between wt 745A and 3Cl8 cells. After selection of neomycin-resistant 745A cells (i.e., clone 141A) and hygromycin-resistant 3Cl8 cells (i.e., clone 341B), the susceptibility of seven somatic hybrid clones (Hy1–7) to IFN-β was investigated. As shown in Supplementary Figure S2, 745A cells complemented the defects of 3Cl8 cells, rendering the somatic hybrids susceptible again to type I IFN. Therefore, the resistant phenotype is due to a loss of function. In this context, we first analyzed the expression of type I IFN receptor chains.

### 2.2. 3Cl8 and 3γR8/ABGR Cell Clones Lack the Expression of the IFNAR-2 Chain

**Figure 4:**
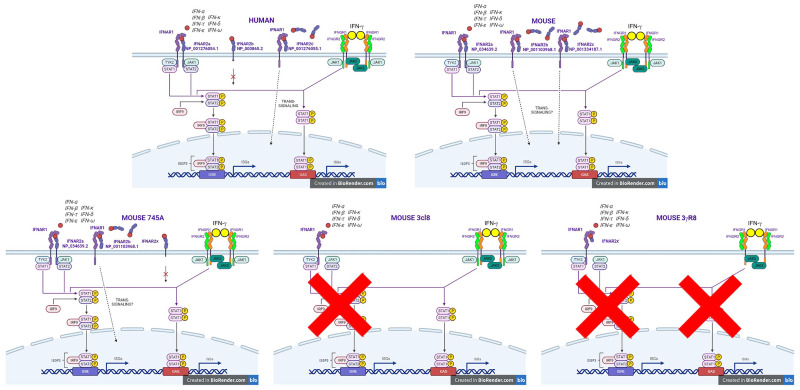
Schematic representation of IFNAR and IFNGR chains and JAK-STAT signal transduction in humans (**top**, **left**), mouse (**top**, **right**) and murine 745A (wt), 3Cl8, and 3γR8/ABGR cell clones (**bottom**). This figure briefly summarizes our results (bottom) and compares them to those reported for human (top left) and mice (top right). The red dots represent all type I IFNs; the yellow dots represent type II IFN (γ), which is a dimer; the type I IFN receptor chains are shown in purple; those for type II IFN are in orange (IFNGR1/IFNGR-α) and green (IFNGR2/IFNGR-β); ISRE: IFN-Stimulated Response Element; GAS: Gamma-Activated Sequence. The red crosses indicate the absence of IFN signal transduction due to the lack of one of the two receptor chains for type I or II IFN in the mutant cell clones. In 745A (wt cells), the soluble receptor chain labeled IFNAR2b in the figure is equivalent to the soluble IFNAR2c chain found in humans, while the non-signaling transmembrane receptor chain IFNAR2x corresponds to the transmembrane non transducing human IFNAR2b chain. Figure adapted from “Mouse ABGR” created in BioRender. Fratini, E. (https://app.biorender.com/illustrations/64c218300803872da053e5f8) accessed on 28 July 2023.

The receptors for type I IFNs are widely distributed and can be found on the surface of all nucleated cells. The IFNAR receptor consists of IFNAR1 and IFNAR2 subunits. IFNAR2 cannot transduce the signal in the absence of IFNAR1 and vice versa [54]. After binding of type I IFN molecules, receptor chains dimerize, thus activating a spectrum of activities [55]. The complexity of ligands, receptor specificity, and signaling components in the murine and human IFN homolog systems is similar [23,56,57,58]. The *ifnar1* gene encodes only one transmembrane isoform in both humans and mice [59,60]. The expression of the *ifnar2* gene produces multiple mRNA transcripts. A nomenclature conflict exists regarding the different IFNAR2 isoforms, as the designations reported in the NCBI sequence database nomenclature (https://ncbi.nlm.nih.gov/protein—accessed on 18 June 2019) conflict with those assigned by the research groups that originally isolated and characterized these chains [12,61,62]. We chose to follow the NCBI database nomenclature. For the sake of clarity, a comparison between the two nomenclatures is provided in Table 1. In humans, IFNAR2 transcripts are translated into three isoforms: IFNAR2a, the longest transmembrane transducing form; IFNAR2b, a transmembrane non-transducing form lacking most of the intracytoplasmic domain required for signaling; and IFNAR2c, the soluble form (Figure 4, top, left side and Table 1). In mice, one transmembrane transducing form and two soluble forms have been described thus far (Figure 4, top, right side and Table 1) [61,62].

Screening of several murine cDNA libraries did not detect a cDNA encoding the murine equivalent of the human transmembrane non-transducing IFNAR2b chain, suggesting that this isoform might be specific to humans [62]. Differential splicing generates two murine transcripts capable of encoding two soluble isoforms. The more abundant (1.5 kb) encodes the complete extracellular domain of the transmembrane transducing form and reads through the splice site at the exon 7-7′ boundary, producing a transcript encoding 12 unique and mostly hydrophobic C-terminal residues (NM_001110498.1). The minor transcript results from skipping exon 8, which encodes the transmembrane region, leading to a frameshift and stop codon, and produces a soluble receptor with 11 unique C-terminal amino acids specific to this isoform (NM_001347258.1). The amino acid sequence for the first 237 amino acids (including the signal sequence) is identical to the transmembrane transducing form [62].

**Figure 5:**
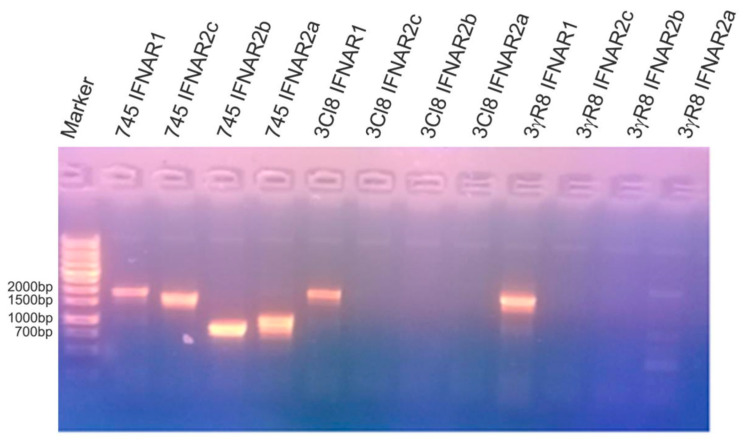
Gel electrophoresis analysis of RT-PCR products related to IFNAR1, IFNAR2a, IFNAR2b and IFNAR2c from the three cell clones 745A, 3Cl8 and ABGR, a subclone of 3γR8. All samples were processed in parallel under identical conditions. Amplification in 745A serves as an internal positive control.

To characterize the expression of IFNAR1 and IFNAR2 in the type I IFN-sensitive and -resistant FLC clones still capable of binding type I IFN [34], the receptor chain transcripts were analyzed. The cDNAs of the IFNAR receptor chains were amplified by RT-PCR, quantified, and analyzed via agarose gel electrophoresis (see Section 4). Amplified fragments of the expected molecular weight corresponding to the PCR products of the IFNAR1 chain from 3Cl8 and ABGR (a subclone of 3γR8 cells with the same phenotype) are present (Figure 5). Conversely, no PCR products for IFNAR2 were detected, indicating that both type I IFN-resistant clones lack IFNAR2 chains (Figure 5). To verify the presence of low-abundance transcripts, nested-PCR was performed on the two resistant cell clones, 3Cl8 and ABGR. PCR products related to IFNAR1 were evident in the resistant clones, while no specific products for IFNAR2 transcripts were amplified in 3Cl8. However, some IFNAR2a- and IFNAR2c-like PCR products were obtained from the ABGR clone (Supplementary Figure S3).

IFNAR1 and IFNAR2 PCR fragments were cloned and aligned with reference sequences from NCBI. The IFNAR1 gene lacks mutations in 745A, 3Cl8, and ABGR although a polymorphism in the fibronectin type III domain of the extracellular region was identified (Histidine 274 instead of Arginine in the murine GenBank sequence, Supplementary Figure S4).

**Figure 6:**
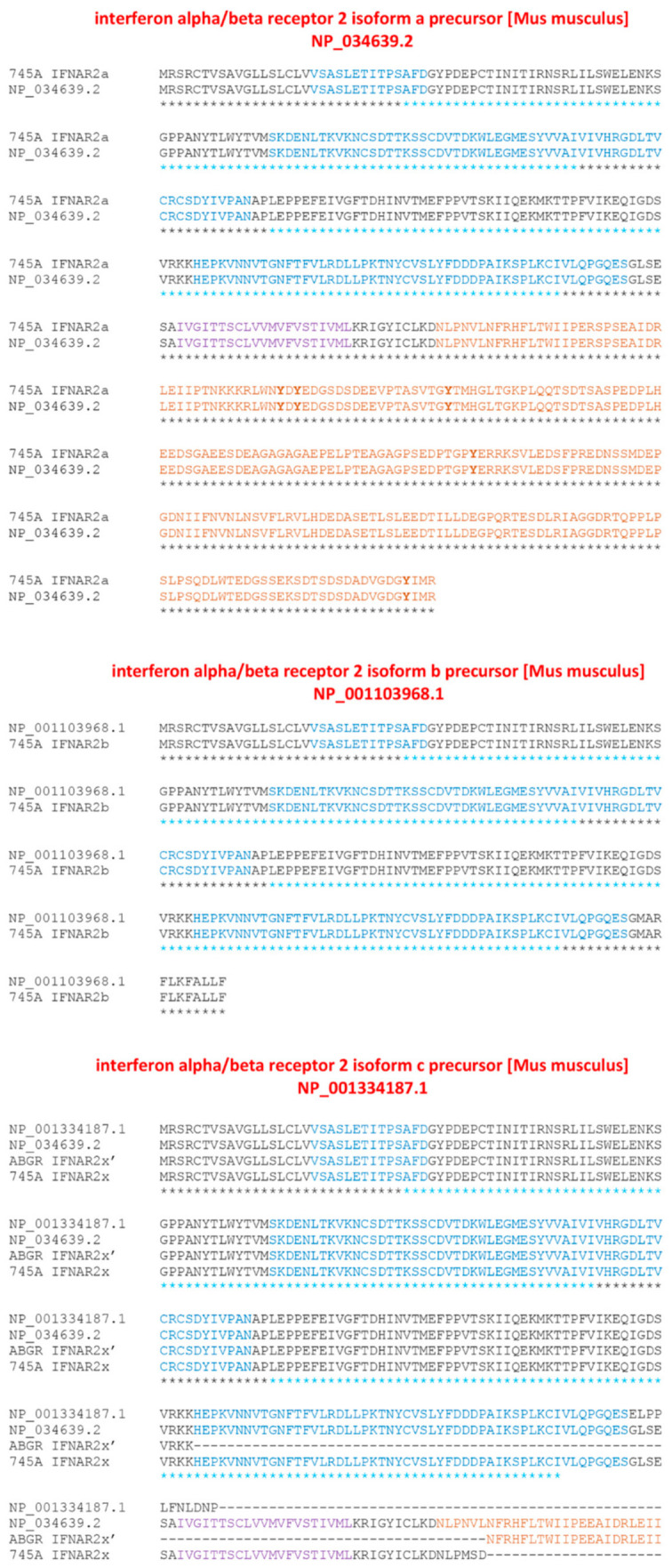
**Top Panel**: Polypeptide sequence alignment of IFNAR2a obtained from 745A cells compared with the reference sequence NP_034639.2 (mRNA, NM_010509.2) from NCBI. Black indicates amino acids coded by the odd exons (1, 3, 5, and 7) corresponding to the extracellular domain. Blue indicates the amino acids coded by the even exons (2, 4, and 6), which are also part of the extracellular domain. Purple indicates the transmembrane region. Orange indicates the intracellular part of the protein. Light blue asterisks denote Fibronectin type III regions. Tyrosine residues are in bold. **Middle Panel**: Polypeptide sequence alignment of IFNAR2b from 745A cells compared with the reference sequence NP_001103968.1 (mRNA, NM_001110498.1) from NCBI. Color coding is as above. **Bottom Panel**: Multiple polypeptide sequence alignment of IFNAR2x and x’ sequences obtained from 745A and ABGR cells, respectively, compared with the reference sequences IFNAR2c (NP_001334187.1, mRNA: NM_001347258.1) and IFNAR2a (NP_034639.2, mRNA: NM_010509.2). Color coding is as above.

Regarding IFNAR2 transcripts, sequencing the 745A-amplified fragment corresponding to IFNAR2a perfectly matches the reference sequence NP_034639.2 (Figure 6, top panel). One of the smaller amplified segments from 745A cells matches the reference sequence NM_001110498.1 (mu IFNAR2b soluble isoform) (Figure 6, middle panel). The other segment was aligned with the mu IFNAR2a isoform (NP_034639.2) and the soluble reference sequence NP_001334187.1 (IFNAR2c), which contains the amino acid derived from the translation of six exons encoding the extracellular domain and an additional 11 amino acids (ELPPLFNLDNP) characteristic of this soluble form. This segment from 745A cells includes all six exons typical of NP_034639.2 (mu IFNAR2a), corresponding to the extracellular protein domain, its transmembrane region, and the first 14 amino acids of the intracellular part followed by 3 additional amino acids. Based on sequence analysis, this transcript of wt cells displays structural features reminiscent of the human transmembrane, non-transducing IFNAR2b isoform. However, we note that this identification is currently based on a single RT-PCR product and subsequent sequence validation. We therefore provisionally define this “atypical” murine isoform as IFNAR2x (Figure 6, bottom panel). At this stage, we interpret IFNAR2x as a putative transcript encoding a transmembrane receptor lacking key intracellular signaling regions, rather than as definitive evidence of a protein product. Regarding ABGR cells, the putative IFNAR2c isoform, derived from the less abundant transcript, includes only the first five exons of the extracellular domain present in the reference sequence NP_001334187.1 (mu IFNAR2c) and an additional 21 amino acids (NFRHFLTWIIPEEAIDRLEII), which are found in the intracellular region of the full-length reference sequence NP_034639.2 (mu IFNAR2a) (Figure 6, bottom panel). Since it lacks a transmembrane domain, this isoform resembles a potential soluble form and is designated muIFNAR2x′. In conclusion the 3Cl8- and ABGR-resistant clones do not produce any transducing IFNAR2 chains but do express a wild-type IFNAR1 receptor chain. Figure 4 (bottom) summarizes these findings schematically.

### 2.3. Complementation Experiments and Sequence Analysis Reveal That the Type I and II IFN-Resistant 3γR8 Cell Clone Does Not Express a Functional Transducing Murine IFNGR β Chain

**Figure 7:**
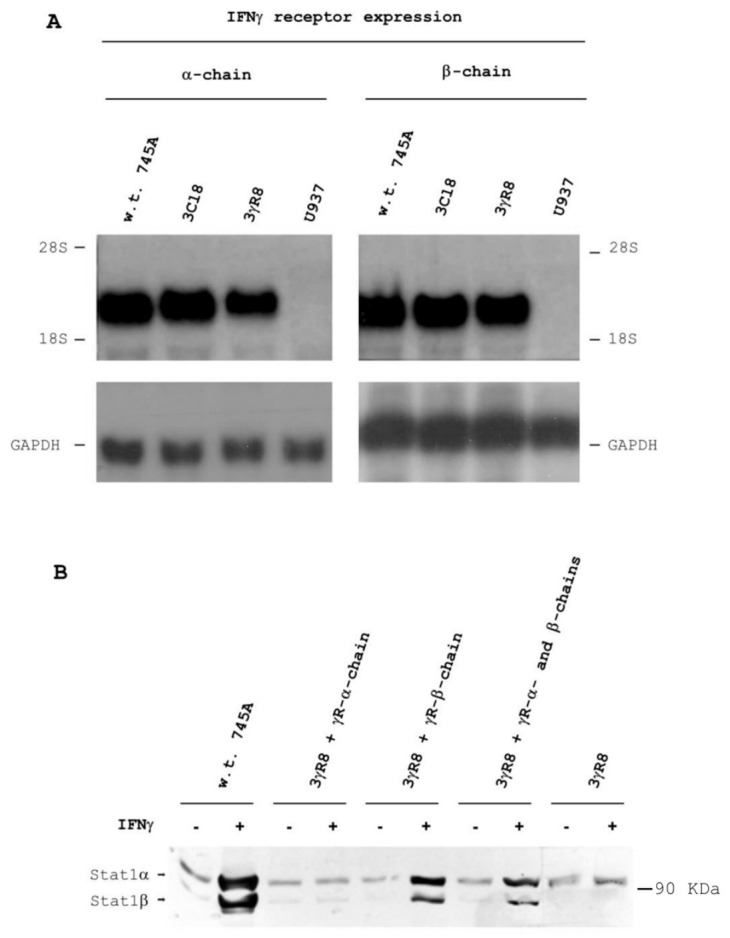
(**A**) Both α and β chain transcripts of IFNGR, the receptor for murine IFN-γ, are expressed in wt 745A, 3Cl8 and 3γR8 FLC clones. Northern blot analysis of IFNGR-α and -β transcripts in RNA extracted from these cells and from human U937 cells (negative control). (**B**) Transient transfection with the β chain, but not the α chain, of IFNGR complements the IFN-γ resistant phenotype of 3γR8 cells. Ten million cells were transiently transfected by electroporation with pHMGC19 (β chain), HMGA 1.2 (α chain) or both DNA as described in Section 4. After 30 h, cells were treated with 500 IU/mL of recombinant mu IFN-γ for 20 h and cell extracts were analyzed by Western blot for STAT1 induction (for densitometric quantification, see Supplementary Materials, Figure S10). Co-transfection with RSV.CAT vector and CAT assay were performed to evaluate transfection efficiency.

To identify the defect that renders the 3γR8 FLC clone IFN-γ-resistant, while still capable of binding IFN-γ [38], a Northern blot analysis was first performed to evaluate the expression of the α and β chains. The presence of a specific transcript for each chain in wt 745A, 3Cl8, and 3γR8 cell clones was observed (Figure 7A). Then, we transiently transfected cells with expression vectors encoding the wild-type α (pHMG19) or β subunit (pHMGA 1.2) of the murine IFN-γ receptor (IFNGR), kindly provided by S. Hemmi and M. Aguet (see Section 4), to assess reversion of the resistant phenotype. Thirty hours after transfection, cells were treated with 500 U/mL of murine IFN-γ, and the steady-state level of STAT1 protein expression was evaluated. As shown in Figure 7B, a marked increase in STAT1 expression was observed in 3γR8 cells transfected with the vector encoding the β subunit of IFNGR and treated with IFN-γ as well as in control wt 745A cells transfected with the empty vector. However, no increase was observed in 3γR8 cells transfected with the vector encoding the α subunit, indicating a defect in the β subunit of the IFNGR in the 3γR8 cells.

Therefore, we sequenced the cDNA of the β chain from the 745A and 3γR8 cells obtained by RT-PCR using the oligonucleotide primers reported in [63] (see Section 4). We identified a single nucleotide deletion at position 646 (starting from the ATG), introducing a stop codon at amino acid 223 in the cDNA of 3γR8, which was not present in the wild-type sequence. The hydrophobic stretch from amino acids 225 to 248 is the putative transmembrane domain, dividing the mature protein into an extracellular domain of 224 amino acids and a cytoplasmic domain of 66 amino acids. This deletion could result in a truncated form of the IFN-γ receptor β, possibly released extracellularly, missing the entire transmembrane and intracellular domains. The absence of the β chain on the cell membrane completely impairs JAK-STAT signal transduction of IFN-γ. Interestingly, the deletion occurs within a stretch of adenines that may be a mutation hotspot (Table 2).

However, we never observed the reversion of the IFN-γ-resistant phenotype over the years. We confirmed the presence of the mutation at the genomic DNA level by amplifying and sequencing the intron containing the putative nucleotide deletion. Figure 4 (bottom) summarizes the results obtained regarding type I and II IFN receptors in FLC clones.

## 3. Discussion

Here, we report the molecular defects that render the 3Cl8 and 3γR8/ABGR FLC clones resistant to murine IFNs. These clones were previously isolated by our group [33,38]. Specifically, 3Cl8 cells are resistant to mu IFN-α and -β [31], whereas 3γR8/ABGR cells are resistant to mu IFN-α, -β, and -γ [36]. The characterized IFN-resistant phenotypes showed that both 3Cl8 and 3γR8 cells bind type I IFNs. However, no transcriptional induction of ISGs occurs after IFN-αβ treatment [33,34,37,38,39,40,41,42]. The 3γR8 cells bind murine IFN-γ. Similarly, no transcriptional induction of ISGs is observed after IFN-γ treatment [33,34,37,38,39,40,41,42]. In wt 745A cells, the JAK-STAT signaling pathway is rapidly activated by type I or II IFNs.

The present work demonstrates that there is no activation of the JAK-STAT pathway after type I IFN treatment of the resistant clones 3Cl8 and 3γR8/ABGR. Specifically, neither JAK1 and Tyk2 nor STAT1 and STAT2 become tyrosine phosphorylated (Figure 1 and Figure 2). Additionally, JAK1, JAK2 and STAT1 are not tyrosine phosphorylated after IFN-γ treatment of 3γR8/ABGR cells (Figure 1 and Figure 2).

The expression of STAT1, STAT2, and STAT3 increases in response to type I and II IFN treatments in susceptible cells. STAT1 and STAT2 are themselves ISGs positively regulated at the transcriptional level via the JAK-STAT pathway. Their increased expression influences and regulates the cell’s susceptibility to other cytokines that utilize STAT1 or STAT3 in their signaling pathways [21,64,65,66]. Accordingly, IFN-β treatment of wt 745A cells increases the expression of STAT1, STAT2, STAT3 and ISG15 after a few hours and IFN-γ increases STAT1, STAT2, and STAT3 expression (Figure 3). The upregulation of STAT1 and STAT2 enhances susceptibility and the kinetics of the transcriptional response of wt FLC to type I or II IFN, contributing to the observed synergism between these IFNs in FLC [35]. IFN-γ treatment of 3Cl8 cells increases STAT expression (Figure 3).

The failure to activate the JAK-STAT pathway suggests that the resistant cells have defects either at the level of JAK kinase functionality or at the level of receptor subunits. These defects ultimately impair signal transduction. Therefore, the expression of IFNAR and IFNGR chains was analyzed in cell clones still capable of binding IFNs [34,38], demonstrating that the defect resides in one of the two IFN receptor chains necessary for coupling ligand binding to JAK-STAT pathway activation.

The IFNAR receptor comprises two transmembrane proteins: IFNAR1 and IFNAR2 (Figure 4) [23]. In the absence of ligand, the receptor chains are separated and only signal upon heterodimerization due to type I IFN binding to both chains.

As described in detail in the Section 2, only one IFNAR1 isoform is present in hu and mu cells, although two splice variants have been identified in some tumor-derived murine cell lines [61]. Conversely, the *ifnar2* gene produces multiple mRNA transcripts that encode three IFNAR2 isoforms: a, b, and c. In humans IFNAR2a is the longest transmembrane transducing form; IFNAR2b is the transmembrane non-transducing isoform, lacking most of the intracellular domain necessary for signaling; IFNAR2c is the soluble form (see Table 1 and Figure 4, top, left side). In mice, one transmembrane transducing form (mu IFNAR2a) and two soluble forms have been described (see Figure 4, top, right side). Differential splicing produces two transcripts encoding the two soluble isoforms [61]. The more abundant transcript encodes the complete extracellular domain of IFNAR2 followed by 12 unique, mostly hydrophobic, C-terminal residues. The less abundant transcript encodes the extracellular domain, lacks the transmembrane domain producing a soluble receptor with 11 unique C-terminal amino acids. To date, no transmembrane non-transducing IFNAR2b isoform has been identified in mice. Figure 4 (top, right panel) summarizes the IFNAR receptor chains described in mice.

In the absence of IFNAR2a, the soluble receptor can bind IFN-α or IFN-β and interact with IFNAR1, inducing partial signaling, although IFNAR1 or IFNAR2 alone does not transduce signals and mediates anti-proliferative responses in primary thymocytes [62]. A soluble mu IFNAR2 has been identified in serum and other fluids, exhibiting both agonistic and antagonistic properties under certain conditions [62]. Studies using recombinant extracellular domains tethered to lipid membranes show that type I IFNs bind to IFNAR2 with a Kd in the nanomolar range, whereas binding to IFNAR1 occurs in the micromolar range. This suggests that IFNAR2 is the high-affinity chain. IFN-α binds with lower affinity, while IFN-β binds more tightly. While human IFNβ conforms to the paradigm, using IFNAR2 as the high-affinity receptor (displaying affinities of ~0.1 nM and 50 nM to IFNAR2 and IFNAR1, respectively), murine IFNβ displays a higher affinity for IFNAR1 than IFNAR2 (12.7 nM vs. ~1600 nM, respectively) [10]. In our previous study we observed a similar affinity constant in wt and 3Cl8 clone via Scatchard plot analysis using iodinated murine natural alpha-beta IFN. Therefore, the coexistence of IFN binding with the complete absence of downstream signaling strongly supports the conclusion that a critical signaling component (IFNAR2) is missing or non-functional in these clones.

We analyzed the sequence of IFNAR receptor transcripts in sensitive and resistant FLC clones. The IFNAR1 chains in all clones appeared intact and did not display mutations, although a polymorphism was identified, All FLC clones, derived from DBA-2 mice, have Histidine (H274) instead of Arginine (R274) in the fibronectin III domain of the extracellular part of IFNAR1 (Supplementary Figure S4). The IFNAR2a chain in the wt 745A clone matches the reference sequence (Figure 6). One of the smaller amplified segments from wt 745A IFNAR2 is also identical to the full-length sequence (NM_001110498.1) of the mu IFNAR2c soluble isoform, corresponding to the IFNAR2*a*′ nomenclature adopted in [12,61,62] (see Section 2, Table 1 and Figure 6). The other segment was compared to both the full-length mu IFNAR2a (NM_010509.2) and the soluble reference sequence mu IFNAR2c (NM_001347258.1), containing six extracellular codifying exons identical to mu IFNAR2a (NM_010509.2), after which the sequence diverges to encode the 11 amino acids (ELPPLFNLDNP) characteristic of this soluble isoform. Interestingly, the segment of the 745A cells contains all six extracellular exons, as well as the transmembrane domain and the first 14 amino acids of the intracellular domain of IFNR2a, followed by 3 unique amino acids (Figure 6). This architecture closely resembles the human transmembrane non-transducing isoform. As previously detailed, no IFNAR2b transmembrane non-transducing chain has been described in mice [62]. In the 3Cl8 and 3γR8 type I-IFN-resistant clones, specific IFNAR2 transcripts were not amplified, indicating that they cannot transduce signals from type I IFNs, likely due to the absence of IFNAR2 expression. More precisely, these data indicate the absence of a functional, transducing IFNAR2 isoform capable of supporting signal propagation. To assess whether low-abundance transcripts were present, we increased the number of amplification cycles. A very low level of a specific transcript was identified only in ABGR lacking the transmembrane codifying sequence but containing the first five exons of the extracellular domain and a fragment encoding 25 amino acids (NFRHFLTWIIPERSPSEAIDRLEII), which exactly matches a portion of the intracellular domain of the full-length IFNAR2a reference sequence NM_010509.2 (Figure 6). This suggests the presence of a complex rearrangement of the wt *ifnar2* gene in this type I IFN-resistant clone. The molecular basis underlying the absence of IFNAR2 transcripts in these resistant clones was not directly investigated in this study and therefore remains to be determined. Several non-mutually exclusive mechanisms could account for this observation, including transcriptional silencing, reduced mRNA stability, or structural alterations affecting the *ifnar2* locus. The detection of aberrant, low-abundance IFNAR2-related transcripts in ABGR cells further supports the possibility of complex genomic or post-transcriptional modifications rather than a simple absence of gene expression.

Importantly, regardless of the underlying mechanism, the lack of functional IFNAR2 expression is sufficient to explain the observed phenotype, as it results in a complete impairment of JAK-STAT activation in response to type I IFNs. Despite the persistence of IFN binding in these cells, no activation of the JAK-STAT pathway is observed, confirming that ligand binding in the absence of a functional IFNAR2 subunit is not sufficient to mediate signal transduction. This observation is consistent with the established requirement for the IFNAR1–IFNAR2 heterodimer to initiate downstream signaling.

In conclusion, the murine 745A wt cell clone produces a full-length IFNAR2a chain, a soluble IFNAR2c isoform, and a third novel isoform, featuring a transmembrane domain such as the human IFNAR2b variant, which likely anchors the protein to the plasma membrane. This isoform should be considered putative, as it is currently supported at the transcript level only, and further studies will be required to determine whether it is expressed as a protein and to define its functional properties. In contrast, the type I IFN-resistant clones express the wt IFNAR1 receptor chain but are unable to transduce signals due to the lack of a functional, transducing IFNAR2 component, even though IFN binding still occurs. Figure 4 (bottom panel) summarizes these findings.

Unlike type I IFNs, which signal as monomers, IFN-γ signals as an antiparallel homodimer [13]. The receptor complex comprises four transmembrane receptor chains: two identical high-affinity (named IFNGR1 or IFNGRα) and two identical low-affinity (named IFNGR2 or IFNGRβ, or accessory factor) chains (see Figure 4). The IFN-γ homodimer directly engages two IFNGR1 chains. IFNGR1 is pre-associated with IFNGR2. Ligand binding induces conformational changes in both receptors. The receptor components are not always co-expressed on the cell surface, but both are required for full signaling activity. Intracellularly, IFNGR1 binds JAK1, and IFNGR2 binds JAK2. JAK1 is necessary for the formation of the signaling complex [13]. Here, we describe the genetic complementation of the IFN-γ resistant phenotype of 3γR8 cells by transient transfection with an expression vector encoding the murine IFNGR β subunit [63], but not the α subunit [67]. Northern blot analysis identified the presence of the respective transcripts in both sensitive and resistant cell clones (Figure 7). Sequencing revealed a single point nucleotide deletion at position 646 in IFNGR2, introducing a stop codon at amino acid 223, and thus predicting a truncated receptor missing the transmembrane and intracellular domains (Table 2). Although direct protein-level evidence is not provided, the predicted structural consequence of this mutation is unambiguous and incompatible with the formation of a functional receptor complex at the plasma membrane. This deletion occurs within a stretch of adenosines, possibly a mutation hotspot. The mutation was confirmed at the genomic DNA level. The IFNGR2 truncated receptor impairs JAK-STAT signaling, accounting for the resistance phenotype. Figure 4, bottom panel, summarizes these results.

## 4. Materials and Methods

### 4.1. Cell Cultures, Interferons and Antibodies

FLC clones grow in suspension in RPMI 1640 (Sigma-Aldrich, St. Louis, MO, USA) medium containing 5% or 10% heat-inactivated fetal bovine serum (FBS), 2 mM L-Glutamine, 100 units/mL penicillin, and 100 ug/mL streptomycin. Cell clones were maintained by diluting confluent cultures between 5 × 10^4^ and 10^5^ viable cells/mL in fresh medium every 3 to 4 days. Cell clones were tested periodically to verify their resistance to the different IFN types, and reversion was never observed.

The cells were treated with the following IFN types: mu IFN-α/β (lot #120), 2.5 × 10^6^ IU/mL, a mix of purified natural mu IFN-αs and IFN-β, kindly provided by Dr. Paola Borghi (Istituto Superiore di Sanità, Rome, Italy); mu IFN-α6, 2 × 10^6^ IU/mL, specific activity 5 × 10^7^ IU/mg of protein; mu recIFN-β (Toray Industries, Inc., Tokyo, Japan), specific activity 3.0 × 10^7^ IU/mg of protein, or Lee Biomolecular Research Laboratories, Inc., San Diego, CA, USA, catalog number 20171, specific activity 5.6 × 10^7^ IU/mg of protein; mu recIFN-γ (ImmunoTools GmbH; Friesoythe, Germany), catalog number #12343534, specific activity 1 × 10^7^ IU/mg of protein.

The following antibodies were used for immunoprecipitation and Western blot: anti-STAT1 mAb (cat. G16920, Transduction Laboratories/BD, catalog number 610116, Lexington, KY, USA); anti-STAT2 cat. sc-950 Santa Cruz Biotechnology, Inc., Dallas, TX, USA); anti-Tyk2 mAb (cat. T20220/L1, Transduction Laboratories); anti-JAK-1 (cat. 06-272, Upstate Biotechnology, Lake Placid, NY, USA); anti-JAK2 (cat. 06-255, Upstate Biotechnology); anti-phospho-tyrosine monoclonal antibody (P-Tyr-100, cat. 9411 Cell Signaling, Danvers, MA, USA); anti-phospho-Stat1 (Y701) rabbit mAb (cat. 7649, Cell Signaling); Anti-phospho-STAT2 (Tyr689) rabbit polyclonal Ab (cat. 07-224, Upstate/MerckMillipore, Darmstadt, Germany); Anti-Phospho-Stat2 (Y689), rabbit polyclonal Ab (cat. ABP-0284 Immunological Sciences, Rome, Italy; anti-Phospho-Stat2 (Y689), rabbit polyclonal Ab (cat. 07-224, Upstate/MerckMillipore. Darmstadt, Germany); anti-β-Actin, mAb (cat. sc-47778, Santa Cruz Biotechnology); anti-β-Tubulin mAb clone Tub2.1 (MP Biomedicals, Santa Ana, CA, USA); anti-ISG15 mAb (cat. sc-166755, Santa Cruz Biotechnology, Dallas, TX, USA.); goat anti-mouse IgG F (ab’)2, polyclonal antibody HRP-conjugate (cat. ADI-SAB-100, Enzo Life Sciences, Inc., Farmingdale, NY, USA); goat anti-rabbit-HRP polyclonal antibody (cat. 170-6515, Bio-Rad, Hercules, CA, USA); goat anti-rabbit IgG Antibody, (H + L) HRP conjugate (cat. AP307P, Merck Millipore, Darmstadt, Germany).

### 4.2. Cellular Extracts and Protein Quantification

Cellular extracts were obtained as previously reported [68]. Briefly, cells were collected and washed once by cold centrifugation at 500× *g* for 5 min using PBS (Phosphate-Buffered Saline, pH 7.4, Sigma-Aldrich, St. Louis, MO, USA). The cell pellets containing 5–10 × 10^6^ cells were lysed in 200–400 µL of lysis buffer (50 mM Tris pH 7.4, 150 mM NaCl, 0.25% deossicolate sodium, 1 mM EDTA, 1 mM EGTA, 1% Triton X-100, 0.5% IGEPAL CA-630, 1 mM sodium orthovanadate, 20 mM sodium fluoride, 1 µg/mL leupeptin and pepstatin A, 2 μg/mL aprotinin and 1 mM phenylmethylsulphonyl fluoride [PMSF], all from Sigma-Aldrich) for 30 min with vortexing every 5–10 min, at +4°. The lysates were centrifuged at 10,000 rpm for 10 min at +4 °C to remove cellular debris and stored at −80 °C. Protein quantification of whole cell lysates was performed using the Bradford assay (Bio-Rad).

### 4.3. Western Blot and Immunoprecipitation

Thirty μg of total protein was resuspended in a volume of 16 μL for each sample and 8 μL of the 3× sample buffer (Tris 65 mM pH 6.8, 10% glycerol, 5% sodium dodecyl sulfate [SDS], 50 mM dithiothreitol [DTT], and 0.05% bromophenol blue). Samples were heated for 5 min at 95–97 °C and resolved by 6 to 13.5% sodium dodecyl sulfate-polyacrylamide gel electrophoresis (SDS-PAGE). Proteins were transferred by electroblotting on 0.45 µm pore size nitrocellulose membranes (Amersham^TM^, GE Healthcare Life Science, Marlborough, MA, USA) using a Mini Trans-Blot apparatus (Bio-Rad). The membranes were washed in TTBS/EDTA (10 mM Tris pH 7.5, 100 mM NaCl, 1mM EDTA, 0.1% Tween 20) and blocked in 3% bovine serum albumin [BSA] and 0.05% sodium azide in TTBS/EDTA for 1 h. Primary antibodies were diluted in 1% BSA TTBS/EDTA and 0.05% sodium azide and incubated with membranes. Afterwards, membranes were washed extensively with TTBS/EDTA. Secondary HRP-conjugated antibodies were applied in TTBS/EDTA, and signals were detected with Enhanced Chemi-Luminescence (ECL) Fast Pico reagents (Immunological Sciences, Rome, Italy) using autoradiographic films or a ChemiDoc XRS and Image Lab software v6.1 (both by Bio-Rad).

For immunoprecipitation, FLC and clones were treated with type I or type II IFN to assess JAKs phospho-tyrosine activation. A pre-clearing step was performed by adding 20 μL of Protein A-Sepharose CL-4B, (Cytiva, Marlborough, MA, USA) to 500 μg of total cell lysate into a final volume of 500 μL by incubating for 30 min with continuous rotation at +4 °C. After pre-cleaning, samples were centrifuged at 425× *g*, for 5 min at +4 °C, and the collected supernatants were incubated for 1 h at +4 °C with 2 μg of anti-JAK1, -JAK2 or -Tyk2 antibodies. Then, samples were added to 20 micrograms of Protein A-Sepharose and incubated for 30 min with continuous gentle agitation at +4 °C. Samples were centrifuged at 425× *g*, for 5 min at +4 °C and the supernatants were discarded. Protein A-Sepharose™ CL-4B containing the immune complexes was washed 4 times with 10 mM NaH_2_PO_4_, 1 mM EDTA, 1 mM EGTA and 20 mM NaF via centrifugation; then, 50 μL of a 2× loading buffer (62.5 mM TRIS pH 6.8, 20% glycerol, 10% SDS, 0.005% bromophenol blue and 0.1 M DTT) was added to the Protein A-Sepharose and the proteins were denatured for 5 min at +95 °C. The samples were centrifuged as already described and the supernatants were analyzed via 7% SDS-PAGE and Western blotting. Signal detection was performed with anti-P-Tyr-100 antibodies and secondary Goat anti-mouse IgG F(ab’)2-HRP conjugated antibodies, followed by ECL detection. A stripping solution was used to remove antibodies from the membrane and to allow reprobing with the same antibodies used for immunoprecipitation. Densitometry was performed on digital files derived from analog acquisition of autoradiographic films, using ImageJ v1.54g (National Institutes of Health, Bethesda MD, USA), and expressed as either phosphoprotein-to-total protein ratio or the ratio of the area of the specific band protein to the area of β-Tubulin.

### 4.4. Cellular Pellet Preparation and RNA Extraction

Cells were seeded at 3 × 10^5^ cells/mL in a 75 cm^2^ flask and incubated for 2 days. Cells were harvested by centrifugation at 500× *g* for 5 min and washed once with PBS and pellets containing 10 × 10^6^ cells per sample were prepared. Total RNA was extracted from the cells (745A, 3Cl8, and 3γR8 or ABGR) using TRIzol Reagent (Invitrogen, Carlsbad, CA, USA) following the manufacturer’s protocol [69]. RNA concentration was quantified using a Nanodrop spectrophotometer (ThermoFisher Scientific, Waltham, MA, USA).

### 4.5. Molecular Cloning Strategy and Sequence Identification of IFNAR Transcripts

To synthesize cDNAs encoding IFNAR proteins, 2 μg of total RNA from clones 745A, 3cl8, 3γR8 and ABGR was reverse-transcribed using random hexamer primers, in a 20 μL reaction volume according to the manufacturer’s instructions (Super-ScriptIII First-Strand Synthesis System for RT-PCR, Invitrogen) [70]. Aliquots of the reverse-transcribed RNA were amplified using Platinum Taq DNA polymerase High Fidelity (Invitrogen) according to the manufacturer’s instructions. The RT-PCR conditions were optimized for each primer pair. PCR primer sequences were designed based on the wild-type sequences available on GenBank for the gene of interest (see Table 3). To amplify the less abundant IFNAR transcripts, nested PCRs were performed (see Table 3 for the primers used). The PCR amplification parameters were an initial denaturation at 94 °C for 5 min, followed by 35 cycles of 94 °C for 1 min, 60 °C for 30 s, and 72 °C for 2 min, with a final extension at 72 °C for 10 min. Full-length cDNAs were cloned into the pCR2.1 vector (Invitrogen). The ligation products were used to transform *E.coli* DH5α strains according to standard protocols [71]. Plasmid DNA was isolated using the QIAprep Plasmid Kit (Qiagen, Hilden, Germany). Sequencing was performed by Microsynth AG (Balgach, Switzerland). The cDNAs were analyzed using the ExPASy Translate tool https://web.expasy.org/translate/ (accessed on 1 July 2019) to determine their open reading frames and predict the amino acid sequences. Polypeptide alignments with reference sequences from NCBI were performed using MSCLE (Multiple Sequence Comparison by Log-Expectation, EMBL-EBI).

### 4.6. Northern Blot Analysis of IFNGR α and β Chains Expression

Total RNA (20 µg), extracted as described, was electrophoresed through denaturing 1.2% agarose gels containing 0.4 M formaldehyde, transferred onto nitrocellulose filters, and hybridized to nick-translated 32P-labeled (1.5 × 10^6^ cpm/mL) probes from pUCA7′.1 and pBlamda1. C19 plasmids, kindly provided by S. Hemmi [63,67], or glyceraldehyde-3-phosphate dehydrogenase (GAPDH) probes. The hybridization conditions were 50 mM sodium phosphate (pH 7.0), 50% formamide, 5× sodium chloride citrate buffer (SSC), 4× Denhardt’s solution, 0.1% sodium dodecyl sulfate (SDS), and 200 µg/mL salmon sperm DNA at 42 °C for 20 h. The filters were washed twice in 1× SSC and 0.1% SDS for 30 min at room temperature and twice in 0.1× SSC and 0.1% SDS for 30 min at 42 °C and then exposed to X-ray film.

### 4.7. Expression Plasmids for α and β Chains of IFNGR and Transient Transfection of Friend Cells

The expression vectors for the α and β chains of murine IFNGR were kindly provided by Michel Aguet and Silvio Hemmi [63,67] and were obtained by inserting the cDNA of the IFNGR α and β subunit into the pHMG backbone [72]. To overexpress the IFNGR α subunit, the IFNGR β subunit, or both chains, exponentially growing subconfluent cells (10^7^) were collected, washed twice in PBS 1×, and resuspended in a final volume of 800 µL of PBS. A total of 30 µg of total plasmid DNA corresponding to10 µg of pRSV-CAT and 20 µg of pHMG-A2.1, pHMG-C19 or 10 µg of both were added to each sample. As control, 20 μg of the pHMG vector plus 10 µg of pRSV-CAT were added to one sample. Cells were loaded into a 0.4 cm electroporation cuvette and transfected using a “Gene-Pulser” apparatus (Bio-Rad) set at 200 V and 500 µF. Cuvettes were then placed on ice for 30 min, and the cells were resuspended in 10 mL of RPMI 1640, 10% FBS. Cells were incubated at 37 °C for 30 h and then stimulated with mu-rec IFN-γ or left untreated for an additional 20 h. After incubation, cells were collected, washed twice in ice-cold PBS, lysed in lysis buffer, and analyzed by Western blot as already described. Transfection efficiency was measured by Chloramphenicol Acetyl Transferase (CAT) assay as previously reported [40].

### 4.8. Sequence Analysis of IFNGR β Subunit cDNA and Genomic DNA Confirmation of Point Mutation

RNA was extracted from cells using RNeasy Kits for RNA Purification (Qiagen), following the manufacturer’s protocol. RNA was recovered in sterile nuclease-free water and stored at −80 °C for later analysis. cDNA synthesis was performed using SuperScript II Reverse Transcriptase (Invitrogen) according to the manufacturer’s instructions. The DNA sequences were obtained by PCR amplification of a portion of the IFNGR β subunit transcript using primers: 5′-catgcggcctttgccactgtggctgccgtc-3′ and 3′-agcagagactggcctggttcacggtg-5′, based on the sequence published in [63]. All samples (745A, 3Cl8, and ABGR) were processed in parallel using the same RNA preparations, reverse transcription reactions, PCR master mix, primer sets, and amplification conditions. Therefore, the experiment inherently includes an internal control across the three clones. The robust amplification of IFNAR2 transcripts in 745A cells using the same reactions demonstrates that the PCR conditions are fully functional. Consequently, the absence of IFNAR2 bands in 3Cl8 and ABGR cannot be attributed to PCR failure or inhibition but rather reflects the absence (or levels below detection) of the corresponding transcripts.

For genomic DNA sequencing, DNA was extracted from cells using the DNeasy Blood & Tissue Kit (Qiagen), following the manufacturer’s protocol. Amplification reactions for direct sequencing were carried out in 100 μL containing 1 μg of genomic DNA, 10 mM Tris–HCl pH 8.3, 50 mM KCl, 1.5 mM MgCl2, 100 mM dNTPs (Pharmacia, Uppsala, Sweden), 0.05 mM primers (Forward: TATAAAACGACGGCCAGTTACAAACYGAGGCACAAC; Reverse; CAGGAAACAGCTATGACCGGATACATTGCTCAAGAG) and 5 U of AmpliTaq Gold (PE Biosystems, Foster City, CA, USA). PCR amplification was performed using a DNA Thermal Cycler 9600 (PE Biosystems) with the following cycle: initial denaturation at 96 °C for 10 min and then 30 cycles of 94 °C for 30 s, 60 °C for 30 s, and 72 °C for 45 s. The PCR product was purified using Microcon 100, Millipore-Merck KGaA, Darmstadt, Germany, following the manufacturer’s instructions. Sequencing was performed with 20–30 ng/mL of the amplicon using the Dye Primer Cycle Sequencing Ready Reaction Kit (PE Biosystems) with M13 primers (-21M13 and M13Rev) labeled with fluorescein or rhodamine. Electrophoretic detection was performed on a 373A DNA Sequencer (PE Biosystems), and sequences were analyzed using sequencing analysis software and Sequence Navigator v7.0 (PE Biosystems).

## 5. Conclusions

Overall, we identified the molecular defect that renders the murine 3Cl8 (type-I IFN-resistant/type II IFN-sensitive) and 3ϒR8/ABGR (type I and II IFN -resistant) cell clones, derived from the wt 745A FLC, resistant to murine type I or murine type I and II IFNs at the level of specific IFN receptor chains blocking the activation of the signal transduction pathways. Notably, our data suggests the existence of a transmembrane, non-transducing mu IFNAR2b isoform in wt FLC and identify a new polymorphism in IFNAR1.

Historically, these IFN-resistant FLC clones have been instrumental in in vitro and in vivo studies of mu IFN biological effects, including modulation of erythroid differentiation [30], antiviral activity [35,36,39,73], and tumor suppression [74,75,76,77,78]. Regarding the antiretroviral effects, both IFN types inhibit the chronic FLV production reversibly in wt FLC, but not in IFN-resistant cells, acting at the level of virus assembly and release [30,33,38]. In vivo passage of 745A or 3Cl8 cells led to the selection of FLV non-producer cells only in wt cells, potentially due to endogenous IFN pressure [75,79]. These clones could further serve to investigate the functional roles of type I and II IFNs and their receptors and to further investigate their role in the regulation of murine Friend virus expression, erythroid differentiation, and extracellular vesicle-IFN-mediated communication [80]. We are currently contacting biobanks to deposit wt and IFN-resistant FLC clones for broader access.

## Figures and Tables

**Table 1:** Nomenclature of murine IFNAR2 receptor chains (in bold) adopted in this paper according to transcript (NM) and protein (NP) reference sequences deposited in NCBI compared to the nomenclature reported in [12,58,61,62]. The length of transcripts encoding IFNAR1 and IFNAR2 proteins are indicated (right).

ID and Nomenclature from NCBI *Mus musculus* (Here Adopted)			Nomenclature from [12,61,62]	Transcript Length
NM_010508.2	NP_034638.2	**IFNAR1**	IFNAR1	7025 bp
NM_010509.2	NP_034639.2	**IFNAR2a**	IFNAR2c	Transcript variant 1 3051 bp
NM_001110498.1	NP_001103968.1	**IFNAR2b**	IFNAR2a	Transcript variant 2 1169 bp (soluble)
NM_001347258.1	NP_001334187.1	**IFNAR2c**	IFNAR2a′	Transcript variant 3 2919 bp (soluble)

**Table 2:** Point mutation in the IFNGR-β subunit of 3γR8 FLC. A single nucleotide deletion (indicated by a hyphen) at position 646 (from the ATG start codon) introduces a stop codon (indicated in bold) at amino acid 223. The hydrophobic stretch from aa 225 to 248 is the putative transmembrane domain dividing the mature protein into an extracellular domain of 224 aa and a cytoplasmic domain of 66 aa.

FLC Clone	Mutation
3γR8	atg…ctgattttgaaaaac-aaaaaatccgaccacatgggctcttgagcaatgtatcctgtcac…tga
745A (wt)	atg…ctgattttgaaaaacaaaaaaatccgaccacatgggctcttgagcaatgtatcctgtcac…tga

**Table 3:** Primer sequences used for each specific gene.

Transcript	Primers	Primer Name
IFNAR 1	5′-AGAAGGAGGAGAATGTGAGC-3′	IFNAR 1 For
	5′-GCTGGCAGCAGAAGCTGG-3′	IFNAR 1 outer Rev
IFNAR 1 Nested	5′-AGAAGGAGGAGAATGTGAGC-3′	IFNAR 1 For
	5′-CTTCAGATAAGTCTGTCTCC-3′	IFNAR 1 inner Rev
IFNAR 2a	5′-GCGATTCAGGTGTGAGAGC-3′	IFNAR 2abc For
	5′-ACACCTGGAGACCACAAGG-3′	IFNAR 2a outer Rev
IFNAR 2a Nested	5′-GCGATTCAGGTGTGAGAGC-3′	IFNAR 2abc For
	5′-CTGAAGAGAAACCCTGTAGG-3′	IFNAR 2a inner Rev
IFNAR 2b	5′-GCGATTCAGGTGTGAGAGC-3′	IFNAR 2abc For
	5′-GGAGTACATTTAGTGTGCTAG-3′	IFNAR 2b outer Rev
IFNAR 2c	5′-GCGATTCAGGTGTGAGAGC-3′	IFNAR 2abc For
	5′-CTTCGTCACTGTCACTGCC-3′	IFNAR 2a outer Rev
IFNAR 2c Nested	5′-GCGATTCAGGTGTGAGAGC-3′	IFNAR 2abc For
	5′-GATGATTTCCAGCCGATCG-3′	IFNAR 2a inner Rev

## Data Availability

The original contributions presented in this study are included in the article/Supplementary Materials. Further inquiries can be directed to the corresponding author.

## Supplementary Materials

The following supporting information can be downloaded at: https://www.mdpi.com/article/10.3390/ijms27135908/s1.
