# Supplementary material for: Interferon Receptor Chain Deficiency in Murine Friend Erythroleukemia Cell Clone Resistant to Type I or Type I and II Interferons
Source: Int J Mol Sci. 2026 Jun 30;27(13):5908. doi: 10.3390/ijms27135908 (PMC13360625; doi:10.3390/ijms27135908)
Supplement: Supplementary file 1 [file ijms-27-05908-s001.zip › ijms-4213295-supplementary.pdf]

International Journal of *Molecular Sciences*

MDPI

Supplementary Materials

Interferon Receptor chain deficiency in murine Friend Erythroleukemia Cell clone resistant to type I or type I and II Interferons

Zulema Antonia Percario[1], Giorgio Mangino[2], Arianna Raponi[1], Emiliano Fratini[3], Gabriele Vaccari[4], Flavia Giannessi[1], Gianna Fiorucci[5], Manuela Cervelli[1], Giovanna Romeo[2] and Elisabetta Affabris[1,*]

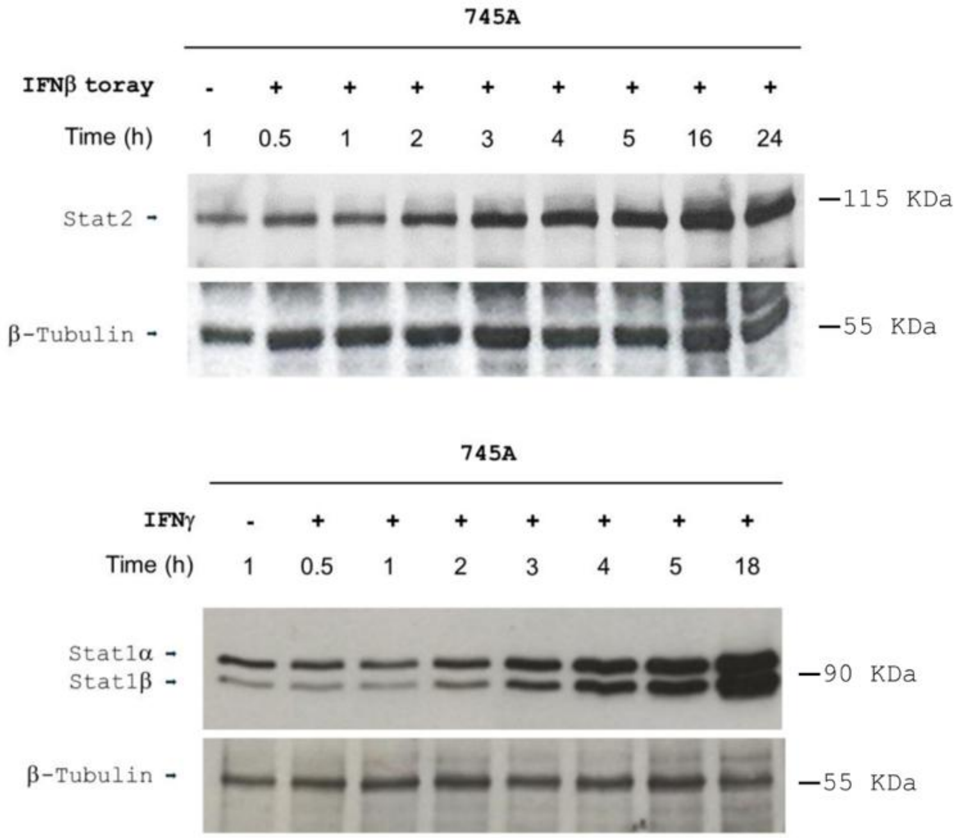

Figure S1. Increasing expression of STAT2 induced by IFN-β and of STAT1 induced by IFN-γ treatment. 745A cells (2.5x10^6 cells/mL for short treatments and 1.5x10^6 cells for 16-to-24-hour treatments) were treated with 500 IU/mL IFN-β or IFN-γ for the indicated times. Immunoblotting was performed with anti-STAT2 or anti-STAT1 antibodies. Anti β-Tubulin was used as a gel loading control. One representative experiment is shown.

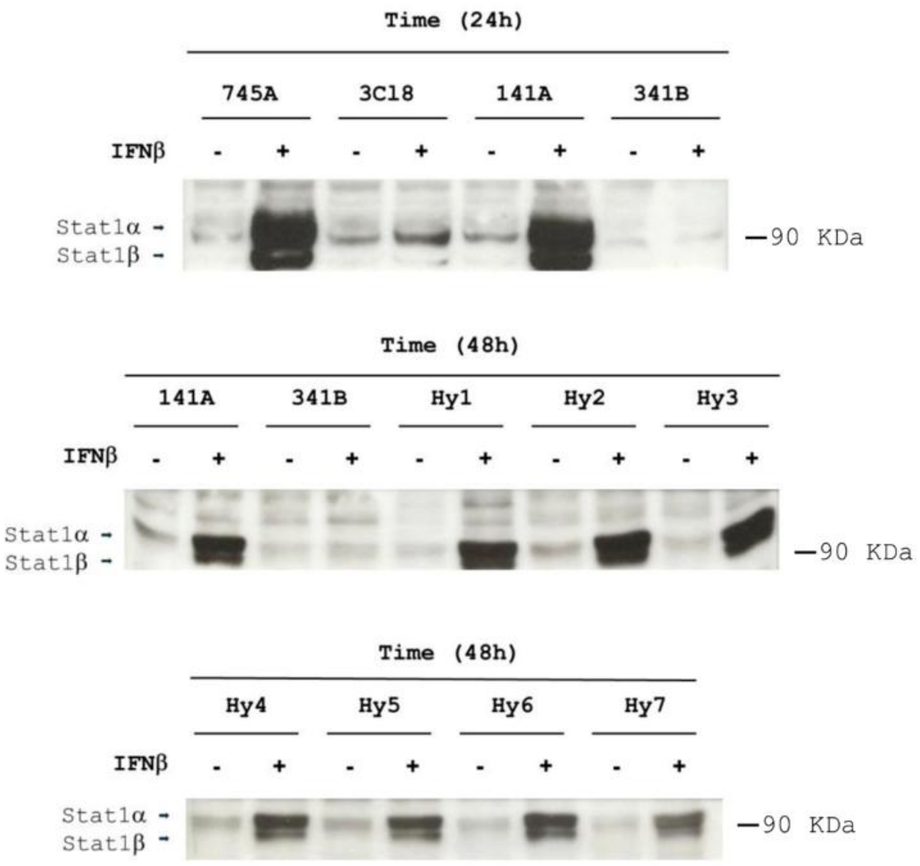

Figure S2. 745A wt cells complement the type I IFN resistance of 3Cl8 cells via somatic hybridization. Cells included 745A (wt), 3Cl8, 141A (a neomycin-resistant 745A-derived clone), 341B (a hygromycin-resistant 3Cl8-derived clone) and hybrid clones from Hy1 to Hy7, treated for the indicated time with 500 IU/mL IFN-β. Western blot analysis was used to evaluate Stat1 expression.

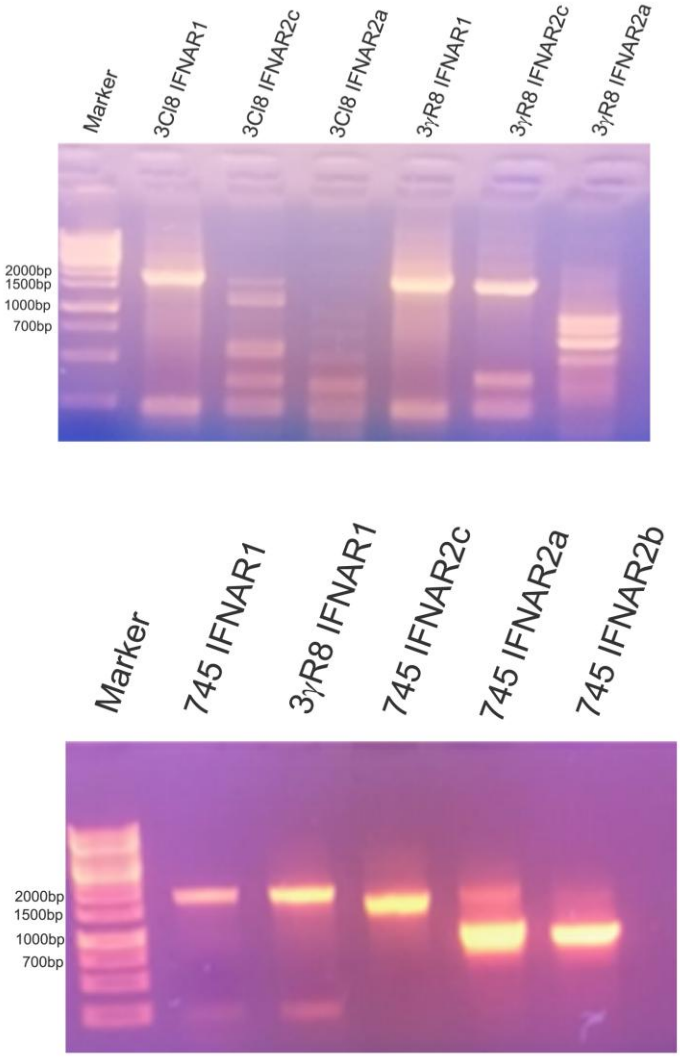

Figure S3. Gel electrophoresis of nested-PCR products related to IFNAR1 and IFNAR2c from 3Cl8 and 3γR8 clones (**upper panel**). PCR products related to IFNAR1, IFNAR2a, IFNAR2b and IFNAR2c from 745A and IFNAR1 from 3γR8 cell clones. (**lower panel**).

```
745A IFNAR1     MLAVVGAAALVLVAGAPWVLPSAAGGENLKPPENIDVYIIDDNYTLKWSSHGESMGSVTF
3Cl8 IFNAR1     MLAVVGAAALVLVAGAPWVLPSAAGGENLKPPENIDVYIIDDNYTLKWSSHGESMGSVTF
3γR8 IFNAR1     MLAVVGAAALVLVAGAPWVLPSAAGGENLKPPENIDVYIIDDNYTLKWSSHGESMGSVTF
NP_034638.2     MLAVVGAAALVLVAGAPWVLPSAAGGENLKPPENIDVYIIDDNYTLKWSSHGESMGSVTF
                ************************************************************

745A IFNAR1     SAEYRTKDEAKWLKVPECQHTTTTKCEFSLLDTNVYIKTQFRVRAEEGNSTSSWNEVDPF
3Cl8 IFNAR1     SAEYRTKDEAKWLKVPECQHTTTTKCEFSLLDTNVYIKTQFRVRAEEGNSTSSWNEVDPF
3γR8 IFNAR1     SAEYRTKDEAKWLKVPECQHTTTTKCEFSLLDTNVYIKTQFRVRAEEGNSTSSWNEVDPF
NP_034638.2     SAEYRTKDEAKWLKVPECQHTTTTKCEFSLLDTNVYIKTQFRVRAEEGNSTSSWNEVDPF
                ************************************************************

745A IFNAR1     IPFYTAHMSPPEVRLEAEDKAILVHISPPGQDGNMWALEKPSFSYTIRIWQKSSSDKKTI
3Cl8 IFNAR1     IPFYTAHMSPPEVRLEAEDKAILVHISPPGQDGNMWALEKPSFSYTIRIWQKSSSDKKTI
3γR8 IFNAR1     IPFYTAHMSPPEVRLEAEDKAILVHISPPGQDGNMWALEKPSFSYTIRIWQKSSSDKKTI
NP_034638.2     IPFYTAHMSPPEVRLEAEDKAILVHISPPGQDGNMWALEKPSFSYTIRIWQKSSSDKKTI
                ************************************************************

745A IFNAR1     NSTYYVEKIPELLPETTYCLEVKAIHPSLKKHSNYSTVQCISTTVANKMPVPGNLQVDAQ
3Cl8 IFNAR1     NSTYYVEKIPELLPETTYCLEVKAIHPSLKKHSNYSTVQCISTTVANKMPVPGNLQVDAQ
3γR8 IFNAR1     NSTYYVEKIPELLPETTYCLEVKAIHPSLKKHSNYSTVQCISTTVANKMPVPGNLQVDAQ
NP_034638.2     NSTYYVEKIPELLPETTYCLEVKAIHPSLKKHSNYSTVQCISTTVANKMPVPGNLQVDAQ
                ************************************************************

745A IFNAR1     GKSYVLKWDYIASADVLFRAQWLPGYSKSSSGSHSDKWKPIPTCANVQTTHCVFSQDTVY
3Cl8 IFNAR1     GKSYVLKWDYIASADVLFRAQWLPGYSKSSSGSHSDKWKPIPTCANVQTTHCVFSQDTVY
3γR8 IFNAR1     GKSYVLKWDYIASADVLFRAQWLPGYSKSSSGSHSDKWKPIPTCANVQTTHCVFSQDTVY
NP_034638.2     GKSYVLKWDYIASADVLFRAQWLPGYSKSSSGSRSDKWKPIPTCANVQTTHCVFSQDTVY
                *********************************.**************************

745A IFNAR1     TGTFFLHVQASEGNHTSFWSEEKFIDSQKHILPPPPVITVTAMSDTLLVYVNCQDSTCDG
3Cl8 IFNAR1     TGTFFLHVQASEGNHTSFWSEEKFIDSQKHILPPPPVITVTAMSDTLLVYVNCQDSTCDG
3γR8 IFNAR1     TGTFFLHVQASEGNHTSFWSEEKFIDSQKHILPPPPVITVTAMSDTLLVYVNCQDSTCDG
NP_034638.2     TGTFFLHVQASEGNHTSFWSEEKFIDSQKHILPPPPVITVTAMSDTLLVYVNCQDSTCDG
                ************************************************************

745A IFNAR1     LNYEIIFWENTSNTKISMEKDGPEFTLKNLQPLTVYCVQARVLFRALLNKTSNFSEKLCE
3Cl8 IFNAR1     LNYEIIFWENTSNTKISMEKDGPEFTLKNLQPLTVYCVQARVLFRALLNKTSNFSEKLCE
3γR8 IFNAR1     LNYEIIFWENTSNTKISMEKDGPEFTLKNLQPLTVYCVQARVLFRALLNKTSNFSEKLCE
NP_034638.2     LNYEIIFWENTSNTKISMEKDGPEFTLKNLQPLTVYCVQARVLFRALLNKTSNFSEKLCE
                ************************************************************

745A IFNAR1     KTRPGSFSTIWIITGLGVVFFSVMVLYALRSVWKYLCHVCFPPLKPPRSIDEFFSEPPSK
3Cl8 IFNAR1     KTRPGSFSTIWIITGLGVVFFSVMVLYALRSVWKYLCHVCFPPLKPPRSIDEFFSEPPSK
3γR8 IFNAR1     KTRPGSFSTIWIITGLGVVFFSVMVLYALRSVWKYLCHVCFPPLKPPRSIDEFFSEPPSK
NP_034638.2     KTRPGSFSTIWIITGLGVVFFSVMVLYALRSVWKYLCHVCFPPLKPPRSIDEFFSEPPSK
                ************************************************************

745A IFNAR1     NLVLLTAEEHTERCFIIENTDTVAVEVKHAPEEDLRKYSSQTSQDSGNYSNEEEESVGTE
3Cl8 IFNAR1     NLVLLTAEEHTERCFIIENTDTVAVEVKHAPEEDLRKYSSQTSQDSGNYSNEEEESVGTE
3γR8 IFNAR1     NLVLLTAEEHTERCFIIENTDTVAVEVKHAPEEDLRKYSSQTSQDSGNYSNEEEESVGTE
NP_034638.2     NLVLLTAEEHTERCFIIENTDTVAVEVKHAPEEDLRKYSSQTSQDSGNYSNEEEESVGTE
                ************************************************************

745A IFNAR1     SGQAVLSKAPCGGPCSVPSPPGTLEDGTCFLGNEKYLQSPALRTEPALLC
3Cl8 IFNAR1     SGQAVLSKAPCGGPCSVPSPPGTLEDGTCFLGNEKYLQSPALRTEPALLC
3γR8 IFNAR1     SGQAVLSKAPCGGPCSVPSPPGTLEDGTCFLGNEKYLQSPALRTEPALLC
NP_034638.2     SGQAVLSKAPCGGPCSVPSPPGTLEDGTCFLGNEKYLQSPALRTEPALLC
                **************************************************
```

Figure S4. Alignment of IFNAR1 (NP_034638.2). Multiple sequence alignment of IFNAR1 from 745A, 3Cl8 and 3γR8 cells, compared with the reference sequence NP_034638.2 (mRNA, NM_010508.2) from NCBI. Black indicates the signal peptide. Green indicates the extracellular part of the protein. Purple indicates the transmembrane region. Ochre indicates the intracellular part of the protein. Light blue asterisks indicate the Fibronectin type III regions. Red box indicates the polymorphism found in the FLC clones.

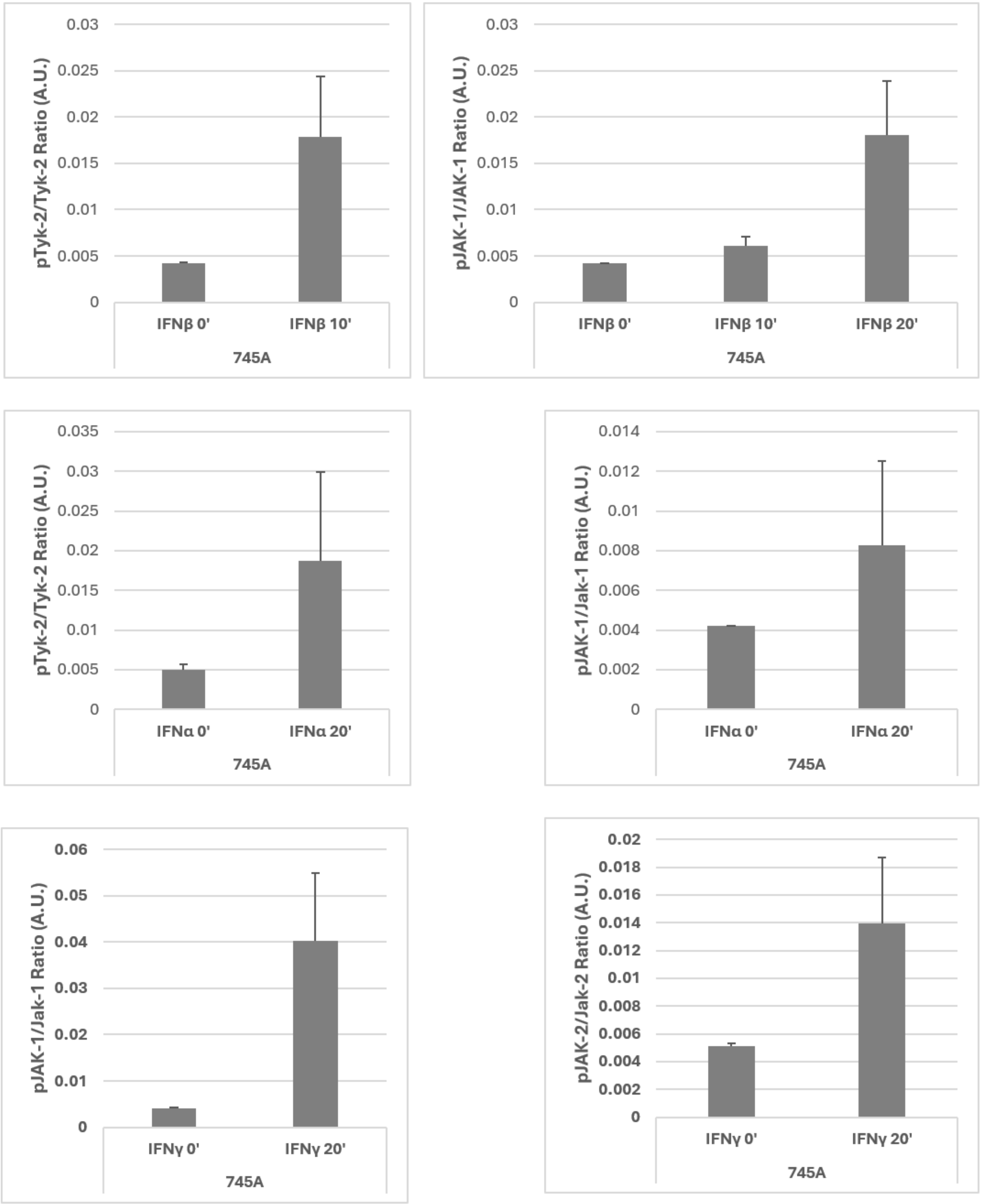

Figure S5. Western blot quantification of Figure 1A. Densitometry was performed on digital files derived from analogical acquisition of autoradiographic films, using ImageJ v1.54g (National Institutes of Health, USA). Data were acquired by measuring band intensity and expressed as a phosphoprotein-to-total protein ratio. Due to the historical nature of the work, Western blot densitometry represents a single representative experiment.

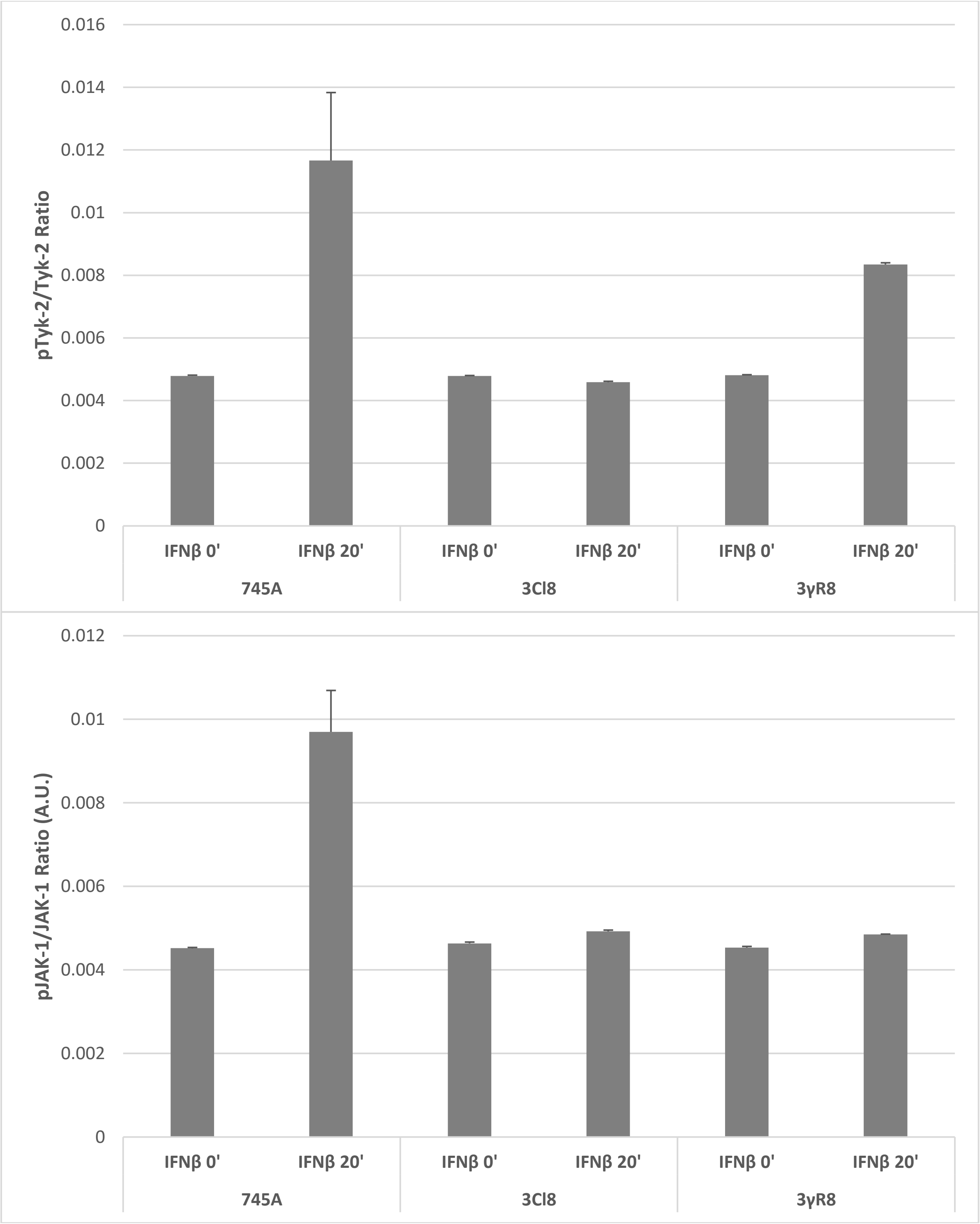

Figure S6. Western blot quantification of Figure 1B. Densitometry was performed on digital files derived from analogical acquisition of autoradiographic films, using ImageJ v1.54g (National Institutes of Health, USA). Data were acquired by measuring band intensity and expressed as a phosphoprotein-to-total protein ratio. Due to the historical nature of the work, Western blot densitometry represents a single representative experiment.

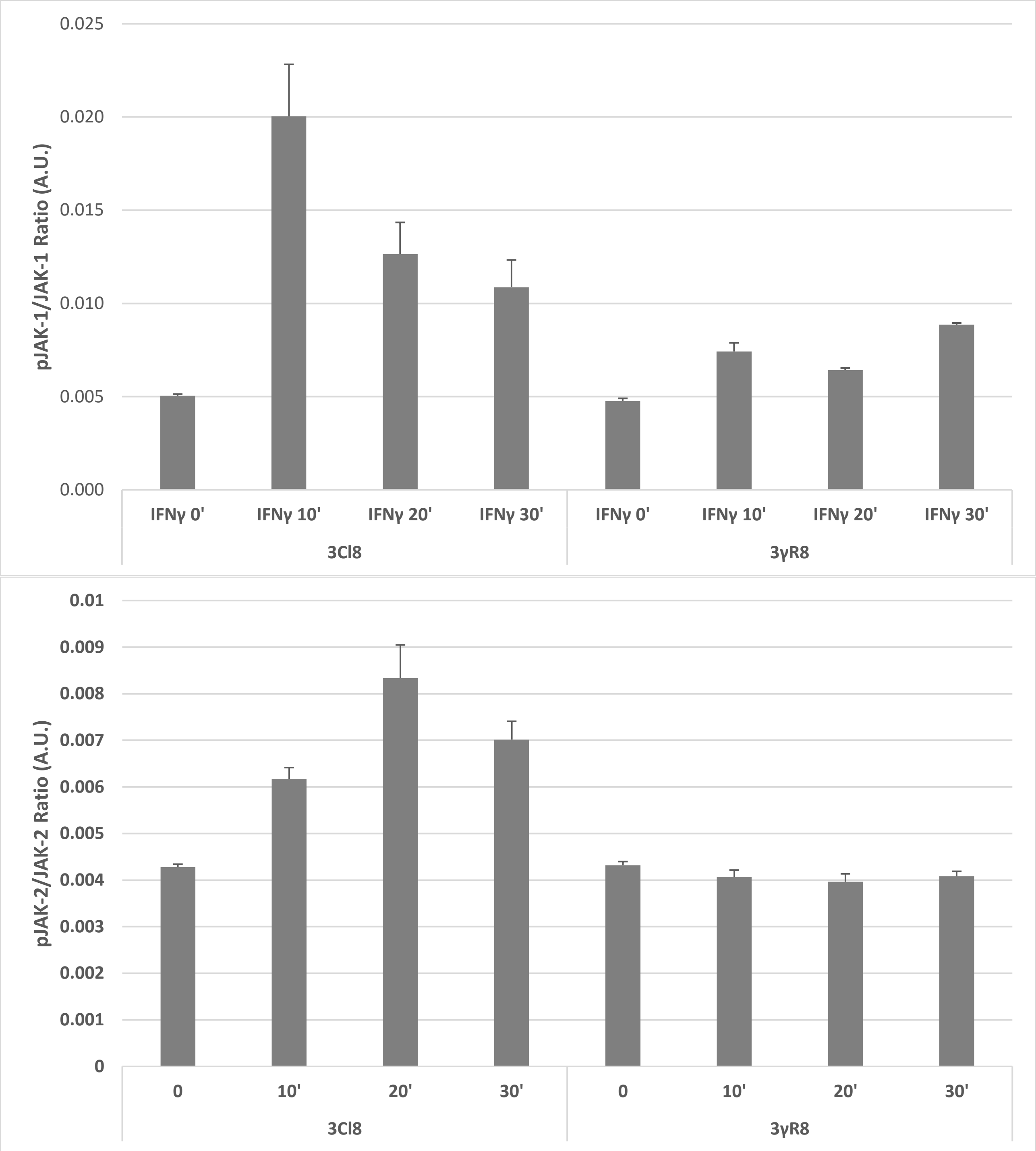

Figure S7. Western blot quantification of Figure 1C. Densitometry was performed on digital files derived from analogical acquisition of autoradiographic films, using ImageJ v1.54g (National Institutes of Health, USA). Data were acquired by measuring band intensity and expressed as a phosphoprotein-to-total protein ratio. Due to the historical nature of the work, Western blot densitometry represents a single representative experiment.

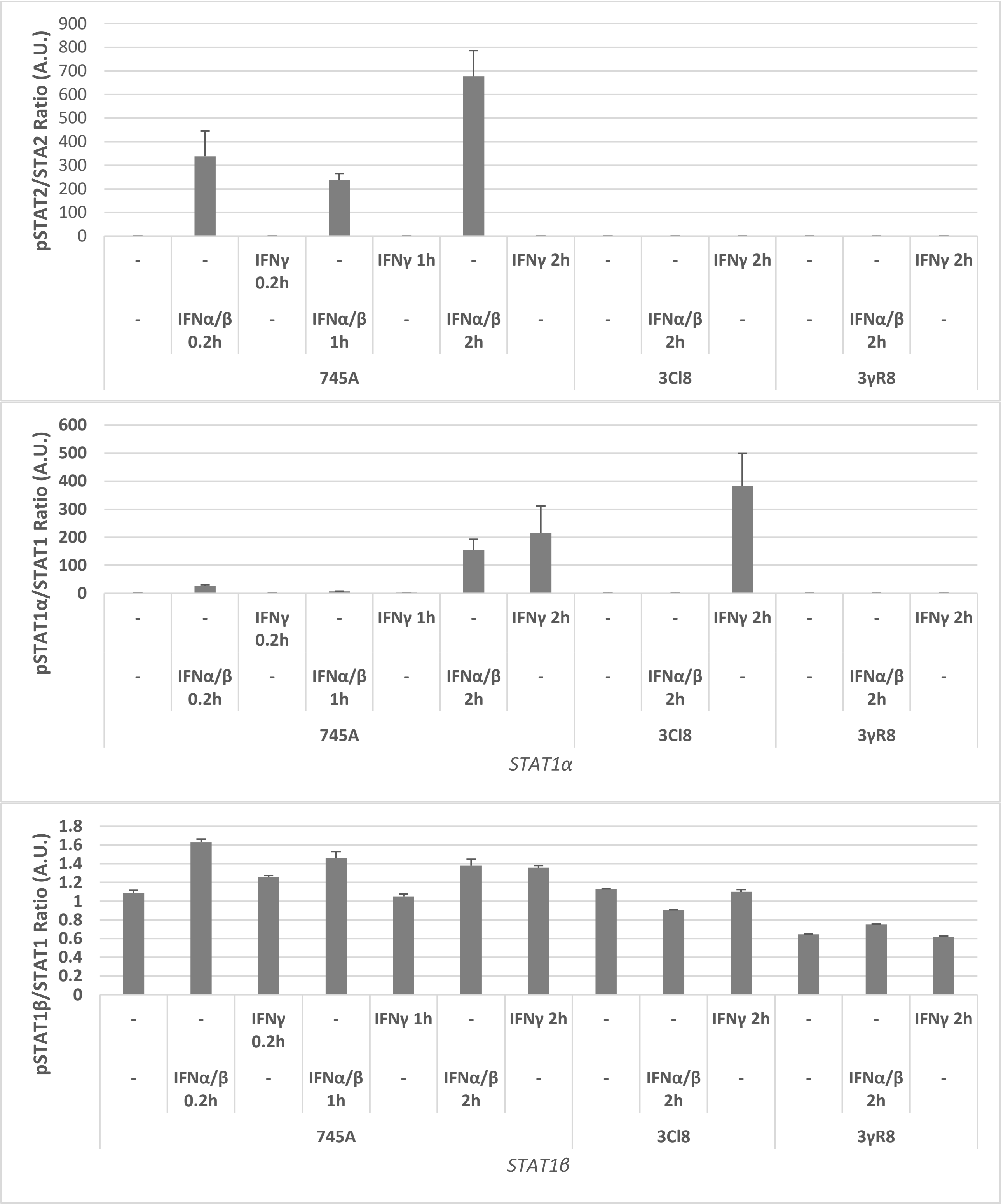

Figure S8. Western blot quantification of Figure 2. Densitometry was performed on digital files derived from analogical acquisition of autoradiographic films, using ImageJ v1.54g (National Institutes of Health, USA). Data were acquired by measuring band intensity and expressed as a phosphoprotein-to-total protein ratio. Due to the historical nature of the work, Western blot densitometry represents a single representative experiment.

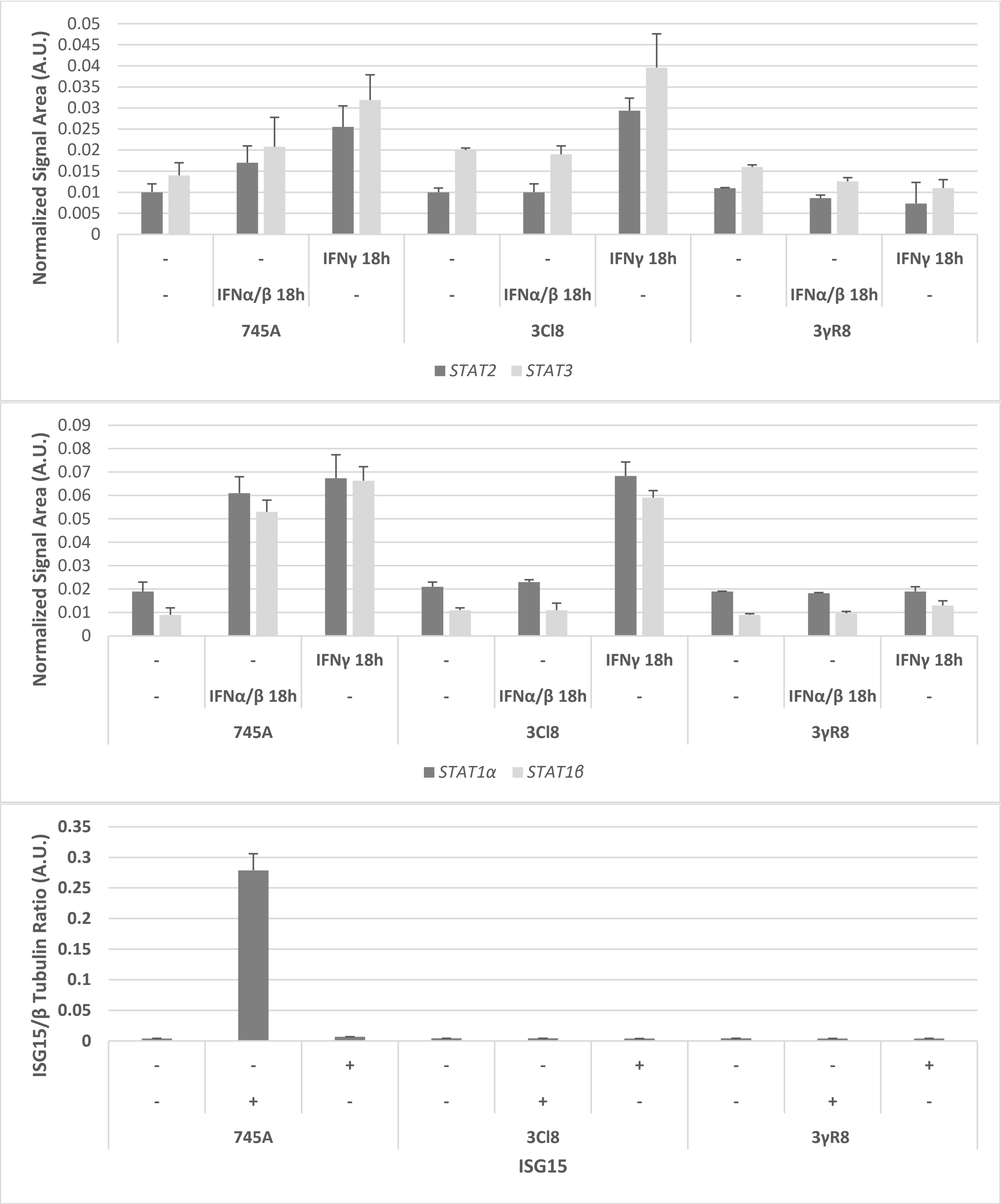

Figure S9. Western blot quantification of Figure 3. Densitometry was performed on digital files derived from analogical acquisition of autoradiographic films, using ImageJ v1.54g (National Institutes of Health, USA). Data were acquired by measuring band area and expressed as the ratio of the area of the specific band protein to the area of β-Tubulin. Due to the historical nature of the work, Western blot densitometry represents a single representative experiment.

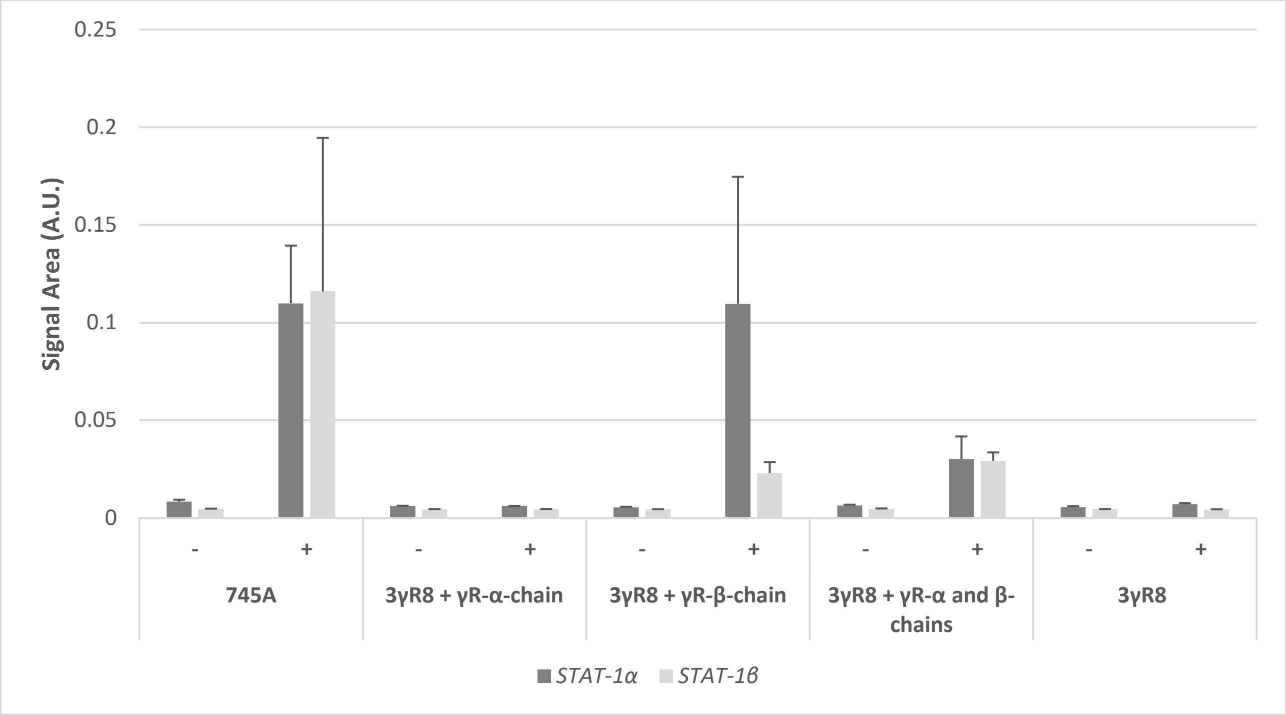

Figure S10. Western blot quantification of Figure 7B. Densitometry was performed on digital files derived from analogical acquisition of autoradiographic films, using ImageJ v1.54g (National Institutes of Health, USA). Data were acquired by measuring band area and expressed as the ratio of the area of the specific band protein to the area of β-Tubulin. Due to the historical nature of the work, Western blot densitometry represents a single representative experiment.
